# Influence of Chronic Electroconvulsive Seizures on Plasticity-Associated Gene Expression and Perineuronal Nets Within the Hippocampi of Young Adult and Middle-Aged Sprague-Dawley Rats

**DOI:** 10.1093/ijnp/pyad008

**Published:** 2023-03-04

**Authors:** Minal Jaggar, Shreya Ghosh, Balaganesh Janakiraman, Ashmita Chatterjee, Megha Maheshwari, Vani Dewan, Brendan Hare, Sukrita Deb, Dwight Figueiredo, Ronald S Duman, Vidita A Vaidya

**Affiliations:** Department of Biological Sciences, Tata Institute of Fundamental Research, Mumbai, India; Department of Biological Sciences, Tata Institute of Fundamental Research, Mumbai, India; Department of Biological Sciences, Tata Institute of Fundamental Research, Mumbai, India; Department of Biological Sciences, Tata Institute of Fundamental Research, Mumbai, India; Department of Biological Sciences, Tata Institute of Fundamental Research, Mumbai, India; Department of Biological Sciences, Tata Institute of Fundamental Research, Mumbai, India; Division of Molecular Psychiatry, Department of Psychiatry and Pharmacology, Yale University School of Medicine, New Haven, Connecticut, USA; Department of Biological Sciences, Tata Institute of Fundamental Research, Mumbai, India; Department of Biological Sciences, Tata Institute of Fundamental Research, Mumbai, India; Division of Molecular Psychiatry, Department of Psychiatry and Pharmacology, Yale University School of Medicine, New Haven, Connecticut, USA; Department of Biological Sciences, Tata Institute of Fundamental Research, Mumbai, India

**Keywords:** PNN, hippocampus, antidepressant, aging, reelin, hippocampal neurogenesis, parvalbumin

## Abstract

**Background:**

Electroconvulsive seizure therapy is often used in both treatment-resistant and geriatric depression. However, preclinical studies identifying targets of chronic electroconvulsive seizure (ECS) are predominantly focused on animal models in young adulthood. Given that putative transcriptional, neurogenic, and neuroplastic mechanisms implicated in the behavioral effects of chronic ECS themselves exhibit age-dependent modulation, it remains unknown whether the molecular and cellular targets of chronic ECS vary with age.

**Methods:**

We subjected young adult (2–3 months) and middle-aged (12–13 months), male Sprague Dawley rats to sham or chronic ECS and assessed for despair-like behavior, hippocampal gene expression, hippocampal neurogenesis, and neuroplastic changes in the extracellular matrix, reelin, and perineuronal net numbers.

**Results:**

Chronic ECS reduced despair-like behavior at both ages, accompanied by overlapping and unique changes in activity-dependent and trophic factor gene expression. Although chronic ECS had a similar impact on quiescent neural progenitor numbers at both ages, the eventual increase in hippocampal progenitor proliferation was substantially higher in young adulthood. We noted a decline in reelin⁺ cell numbers following chronic ECS only in young adulthood. In contrast, an age-invariant, robust dissolution of perineuronal net numbers that encapsulate parvalbumin⁺ neurons in the hippocampus were observed following chronic ECS.

**Conclusion:**

Our findings indicate that age is a key variable in determining the nature of chronic ECS-evoked molecular and cellular changes in the hippocampus. This raises the intriguing possibility that chronic ECS may recruit distinct, as well as overlapping, mechanisms to drive antidepressant-like behavioral changes in an age-dependent manner.

**Significance Statement**

Electroconvulsive therapy is often the treatment of choice for treatment-resistant geriatric depression given its rapid-onset therapeutic benefits. Studies that aim to investigate the molecular, cellular, and behavioral mechanisms targeted by electroconvulsive seizure (ECS) have predominantly used young adult animals. It remains unclear whether ECS influences the middle-aged brain in a similar manner to effects in young adulthood. Here, we sought to compare the effects of chronic ECS on neurotrophic, neurogenic, and extracellular matrix (ECM)-associated changes in young-adult and middle-aged Sprague-Dawley rats. Although ECS evoked robust antidepressant-like behavioral effects at both ages, we noted unique as well as overlapping, age-dependent changes in neurotrophic factor expression, hippocampal neurogenesis, ECM-associated gene expression, and reelin^+^ cells and perineuronal net numbers. This raises the intriguing possibility that chronic ECS may recruit both common and distinctive mechanisms in young adulthood and in middle-aged life to drive the robust ECS-evoked antidepressant-like behavioral changes.

## INTRODUCTION

Electroconvulsive seizure therapy (ECT) is a fast-acting, non-pharmacological antidepressant treatment often used for treatment-resistant depression (Rubin et al., 1991; Lima et al., 2013). ECT is a treatment of choice for patients, both young adult and geriatric, who do not respond to pharmacological antidepressants and is suggested to be particularly effective in geriatric depression (Kramer 1987; Heijnen et al., 2019; Dominiak et al., 2021), given that treatment resistance tends to have a higher incidence in older patient cohorts (Thomas et al., 2013; Lin et al., 2022). Preclinical studies indicate that chronic electroconvulsive seizures (ECS) evoke enhanced trophic factor signaling (Nibuya et al., 1995; Dias et al., 2003; Newton et al., 2003a; Altar et al., 2004b), adult hippocampal neurogenesis (Madsen et al., 2000; Malberg et al., 2000; Scott et al., 2000), dendritic and synaptic remodeling (Chen et al., 2009; Zhao et al., 2012), mossy fiber sprouting (Vaidya et al., 1999), and an increase in the volume of the CA1 hippocampal subfield (Kaae et al., 2012; Chen et al., 2020). Although clinical studies report greater efficacy of ECT in geriatric patients (Jenike 1989; Gomez and Gomez, 1992), preclinical studies are predominantly restricted to young adulthood (Duman and Vaidya 1998; Bouckaert et al., 2014) and do not provide insights into whether similar mechanisms operate at the molecular and cellular level when ECS is administered to older animals.

The aging hippocampus progressively loses the ability to exhibit adaptive plasticity-like responses and is associated with neurotrophic and neurogenic decline (Bettio et al., 2017; Babcock et al., 2021). Here, we sought to assess and compare the influence of chronic ECS on mood-related behavior, plasticity-associated gene expression, neurotrophic and neurogenic changes in the hippocampus, as well as extracellular matrix (ECM)-associated moieties, namely the perineuronal nets (PNNs), and enzymatic machinery linked to PNN synthesis/dissolution in young adult and middle-aged rats. We assessed changes in the ECM, given reports that antidepressants target the PNNs (Maya Vetencourt et al., 2008; Venturino et al., 2021), chondroitin sulphate proteoglycan-rich ECM structures deposited predominantly around parvalbumin (PV^+^) inhibitory interneurons (Härtig et al., 1992; Fawcett et al., 2019). PNN formation is linked to critical period closure, and PNN dissolution is associated with the reopening of a critical period plasticity-like state (Umemori et al., 2018; Reha et al., 2020). We examined the influence of ECS on hippocampal expression of reelin, a secreted glycoprotein that directly influences synaptic and structural plasticity (Lee and D’Arcangelo, 2016; Jossin, 2020). Our results highlight that ECS evokes both unique and overlapping molecular signatures, with neurogenic changes robustly attenuated within the middle-aged hippocampal milieu. Further, we found a significant reduction in PNN numbers within the hippocampal subfields following ECS that is independent of the age of onset of treatment, whereas a reduction in hippocampal reelin^+^ cell number was restricted to young adulthood. These findings provide novel evidence that the effects of ECS at the molecular, cellular, and behavioral levels show both distinct and common features in young adult and middle-aged rats.

## MATERIALS AND METHODS

### Animals

Male Sprague-Dawley rats (young adult: 2–3 months; middle-aged: 12–13 months) bred in the Tata Institute of Fundamental Research (TIFR) animal facility and maintained on a 12-hour-light/-dark cycle (7 am–7 pm) with ad libitum access to food and water were used for all experiments. Experimental procedures followed the guidelines of the Committee for the Purpose of Control and Supervision of Experiments on Animals, Government of India and were approved by the TIFR animal ethics committee (TIFR/IAEC/2017-2).

### Chronic ECS Paradigm

Young adult and middle-aged rats received a series of 1 ECS or sham treatment per day for 7 consecutive days. Bilateral ECS treatment was administered via spring-loaded ear clip electrodes (ECT unit, UGO Basile, Comerio, Italy), and sham treatment involved the application of ear clip electrodes without electrical stimulation. Chronic ECS parameters for each ECS treatment were as follows: current strength: 70 mA; duration: 0.5 seconds; frequency: 100 pulses/s; pulse width: 0.9 milliseconds. Behavioral assays or brain tissue harvesting for further processing was performed 2 hours post the last ECS treatment on the seventh day (Dias et al., 2003). The choice of the older age cohort for chronic ECS was based on preliminary experiments that determined minimal mortality following chronic ECS in the middle-aged (12 months) Sprague-Dawley rats.

### BrdU Labeling

To examine adult hippocampal progenitor proliferation within the dentate gyrus (DG) hippocampal subfield, sham and chronic ECS groups received the mitotic marker bromodeoxyuridine (BrdU; 200 mg/kg; Sigma-Aldrich, Missouri, USA) intraperitoneally 1 hour after the final ECS treatment and were killed 2 hours post BrdU administration.

### Modified Forced Swim Test (FST)

The FST performed to assess despair-like behavior included a pre-swim (15 minutes) in a cylindrical tank (50-cm height × 21-cm diameter) filled with water (24°C, height: 35 cm) on day 1. On day 2, animals were placed in the tank for 6 minutes and allowed to freely swim within the tank for the first 1 minute before manual assessment from video recordings of the time spent immobile for the remaining 5 minutes.

### Quantitative Real-Time Polymerase Chain Reaction (qPCR) Analysis

Hippocampal RNA was extracted using TRI reagent (Sigma-Aldrich) and reverse transcribed using the PrimeScript 1st strand cDNA Synthesis Kit (Takara Bio, Shiga, Japan). cDNA was subjected to qPCR using the CFX96 qRT-PCR system (Bio-Rad, California, USA). The complete list of primer sequences is in supplementary Table 1. Hypoxanthine guanine phosphoribosyl transferase (*Hprt*) was used for normalization of qPCR data. Data analysis was performed using the ΔΔ^Ct^ method as previously described (Bookout and Mangelsdorf 2003) and is represented as fold change ± SEM. The rationale for the chosen candidate genes can be found in supplementary Table 3.

### Immunohistochemical Analysis

Animals were killed 3 hours after the final ECS/sham treatment by transcardial perfusion with 4% paraformaldehyde. Coronal sections (50 μm; VT1000S vibratome, Leica, Germany) spanning the rostro-caudal extent of the hippocampus were subjected to immunofluorescence staining for the mitotic marker BrdU, for the PNN marker, biotinylated plant lectin, Wisteria Floribunda Agglutinin (WFA) or the ECM-associated glycoprotein, reelin. Hippocampal sections were subjected to double immunofluorescence analysis for sex-determining region Y (SRY)-related HMG box 2 (Sox2) and glial fibrillary acidic protein (GFAP) to identify Sox2⁺-GFAP⁺ quiescent neural progenitors (QNPs) within the subgranular zone (SGZ) of the DG subfield and PV-WFA double-positive interneurons within the distinct hippocampal subfields.

For BrdU immunohistochemistry, every sixth section (12 sections per animal) was processed as previously described (Malberg et al., 2000). Briefly, following DNA denaturation and acid hydrolysis, sections were incubated overnight with mouse anti-BrdU (B2531, Sigma-Aldrich; 1:500), subjected to washes, and incubated with a secondary anti-mouse antibody. Six free-floating hippocampal sections (250 μm apart) per animal were processed as follows. For PNN staining, the sections were incubated with biotinylated WFA (B1355, Vector Laboratories, California, USA; 1:250) overnight followed by incubation with 488 Alexa-Fluor conjugated donkey anti-streptavidin (S11223, Invitrogen, Massachusetts, USA; 1:500). For Sox2⁺-GFAP⁺ immunofluorescence, sections were incubated overnight with primary antibody cocktail of goat anti-Sox2 (SC-17320, Santa Cruz, Texas, USA; 1:1000) and rabbit anti-GFAP (G9269, Sigma-Aldrich; 1:2000) followed by incubation with 488 Alexa-Fluor conjugated donkey anti-goat (SA5-10086, Invitrogen; 1:500) and 555 Alexa-Fluor conjugated donkey anti-rabbit (A31572, Invitrogen; 1:500) secondary antibodies. For PNN and PV double immunofluorescence, sections were incubated overnight with rabbit anti-parvalbumin (ab11427, Abcam, Cambridge, UK; 1:1000) and biotinylated WFA followed by incubation with 488 Alexa-Fluor conjugated donkey anti-streptavidin (S11223, Invitrogen; 1:500) and 555 Alexa-Fluor conjugated donkey anti-rabbit (A31572, Invitrogen; 1:500). To estimate the number of reelin⁺ cells, sections were incubated overnight with mouse anti-reelin (MAB5364, Sigma Aldrich; 1:1000) followed by incubation with biotinylated horse anti-mouse (BA2000, Vector Laboratories; 1:500). Sections were incubated with Avidin-Biotin complex (PK-6100, Vector Laboratories) in 0.1M phosphate buffer for 90 minutes and visualized with diaminobenzidine (D4293, Sigma Aldrich) staining.

### Cell Counting Analysis

A modified unbiased stereological approach was used to estimate the number of BrdU⁺ cells. The number of BrdU⁺ cells per dorsal and ventral DG was individually summed and multiplied by section periodicity to estimate the total number of BrdU⁺ cells in the dorsal and ventral DG subfield respectively. The total number of reelin⁺ cells and WFA^+^ PNNs were counted per animal per hippocampal subfield (Axioskop-2 Plus microscope; Zeiss, Jena, Germany) and divided by the number of sections to obtain an average number of cells per section within the respective hippocampal subfield. To determine percent colocalization of PV^+^ cells with WFA, a minimum of 50 PV⁺ cells were analyzed per animal using z-plane sectioning (0.5-μm steps) in the DG, CA1, and CA3 subfields (Zeiss Axio Imager M2). To determine Sox2⁺-GFAP⁺ cell numbers, double-positive cells were confirmed using z-plane sectioning (0.5 μm) steps at 40× magnification (Zeiss Axio Imager M2) within the SGZ of the dorsal DG subfield.

### Statistical Analysis

Unpaired, 2-tailed Student’s *t* test was used for 2-group experiments and 2-way ANOVA analysis for 4-group experiments, with the 2 variables of chronic ECS and age to determine main and interaction effects. Bonferroni post hoc tests were performed when the 2-way ANOVA analysis indicated a significant chronic ECS by age interaction effect. For gene expression analysis, false discovery rate (FDR) was carried out as per the Benjamini-Hochberg method, and false discovery rate–corrected *P* values have also been reported. Welch’s correction was applied when variances significantly varied between the treatment groups. GraphPad Prism 8 (GraphPad Software Inc., USA) was used to perform the statistical analyses. Data are expressed as mean ± SEM, and statistical significance was set at *P *< .05.

## RESULTS

### Chronic ECS Regulates Behavioral Despair and Hippocampal Activity-Dependent Gene Expression

**Figure 1.**
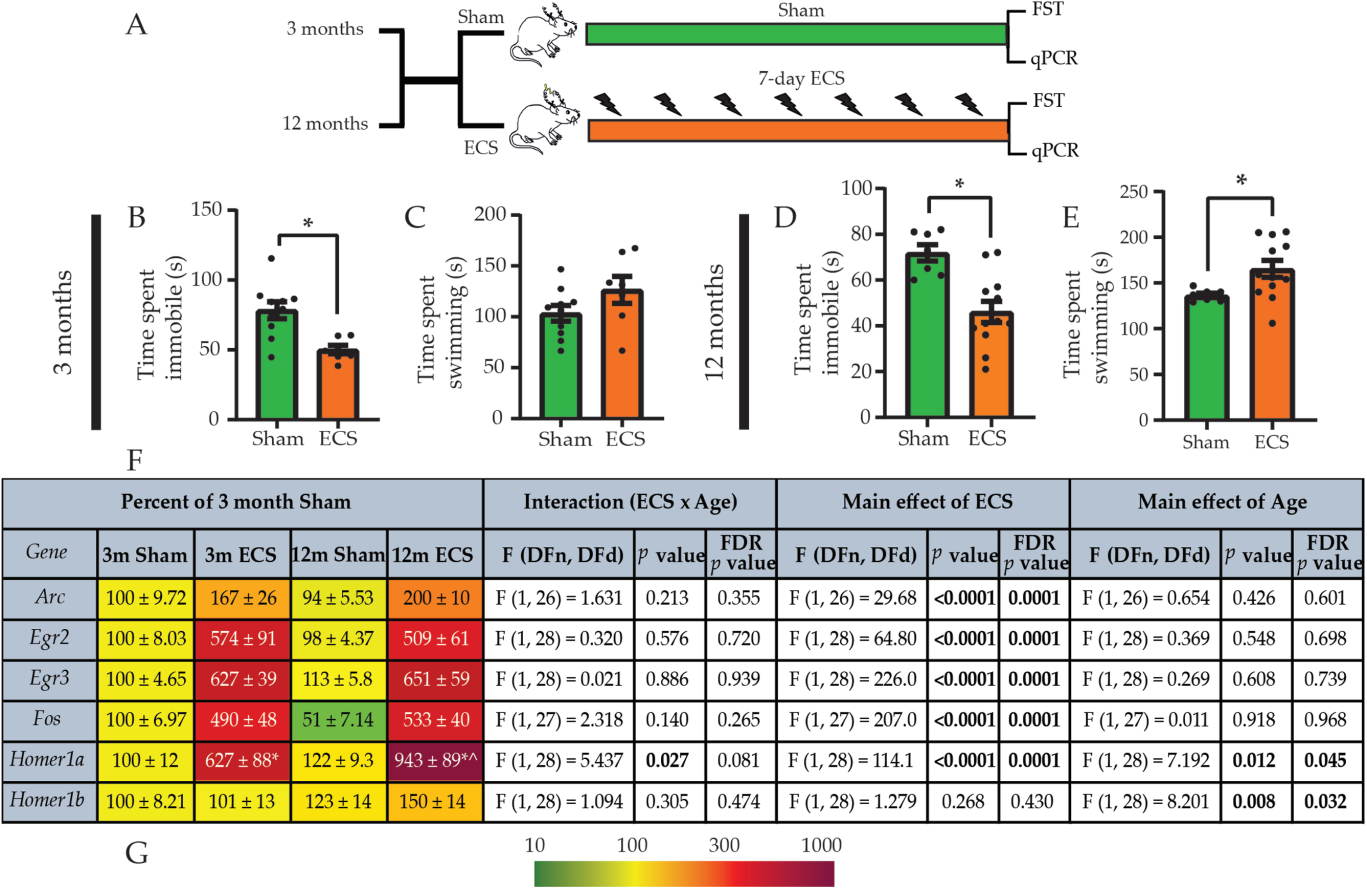
Chronic electroconvulsive seizure (ECS) regulates behavioral despair and hippocampal activity–dependent gene expression in young adult and middle-aged rats. (A) Shown is a schematic depicting the experimental paradigm used to test the effects of chronic ECS on behavioral despair, as assessed by the forced swim test (FST), and on the hippocampal expression of activity-dependent genes, as assessed by quantitative polymerase chain reaction (qPCR), in young adult and middle-aged rats. FST and qPCR analysis were performed on independent experimental cohorts. Chronic ECS led to a significant decrease in immobility time in young adult rats (B) but did not alter the time spent swimming (C). (D) Seven days of chronic ECS led to a significant decrease in immobility time and (E) a significant increase in time spent swimming in middle-aged rats. Results of the behavioral analysis are expressed as mean ± SEM; *p < .05 compared with age-matched, sham-treated controls (unpaired Student’s *t* test; for 3 months, n = 11 for sham, n = 7 for chronic ECS; for 12 months, n = 9 for sham, n = 14 for chronic ECS). (F) Shown are normalized hippocampal gene expression levels for sham- and chronic ECS–treated young adult and middle-aged rats (for young adult and middle-aged animals: n = 8/group), represented as a percent of the young adult sham-treated controls. Heat maps indicate the degree of regulation, with upregulated genes represented in red and downregulated genes represented in green (key, G). Two-way ANOVA analysis for the independent variables of chronic ECS and age are reported as F values, *p* values, and false discovery rate–corrected *p* values for interaction between chronic ECS and age as well as main effects for chronic ECS and age. Results of the qPCR analysis are expressed as mean ± SEM. Bonferroni post hoc group comparisons were performed when a significant 2-way ANOVA interaction was noted between chronic ECS and age, *p < .05, compared with age-matched sham groups, and ^*p *< .05 compared with young adult chronic ECS cohorts.

Chronic ECS evoked a significant reduction in immobility time in both young adults (Figure 1B) and middle-aged rats (Figure 1D) on the FST (Figure 1A; supplementary Table 2). We observed that chronic ECS led to a significant increase in active swimming time only in the middle-aged cohort (Figure 1C and E). Chronic ECS upregulated the expression of several activity-dependent genes, namely activity-regulated cytoskeleton-associated protein (*Arc*), early growth response 2, 3 (*Egr2*, *Egr3*), proto-oncogene *c-fos*, and Homer scaffold protein 1a (*Homer1a*), at both ages (Figure 1F and G; supplementary Figure 1A and B). We observed significant main effects of chronic ECS for the regulation of gene expression of *Arc*, *Egr2*, *Egr3*, and *Fos*. Two-way ANOVA analysis indicated a significant chronic ECS and age interaction, with a greater magnitude of *Homer1a* mRNA upregulation in the middle-aged chronic ECS cohort. Collectively, our results indicate the influence of chronic ECS on despair-like behavior, and the regulation of activity-dependent gene expression did not vary substantially across ages examined.

### Chronic ECS Alters Hippocampal Trophic Factor Gene Expression in an Age-Dependent Manner

**Figure 2.**
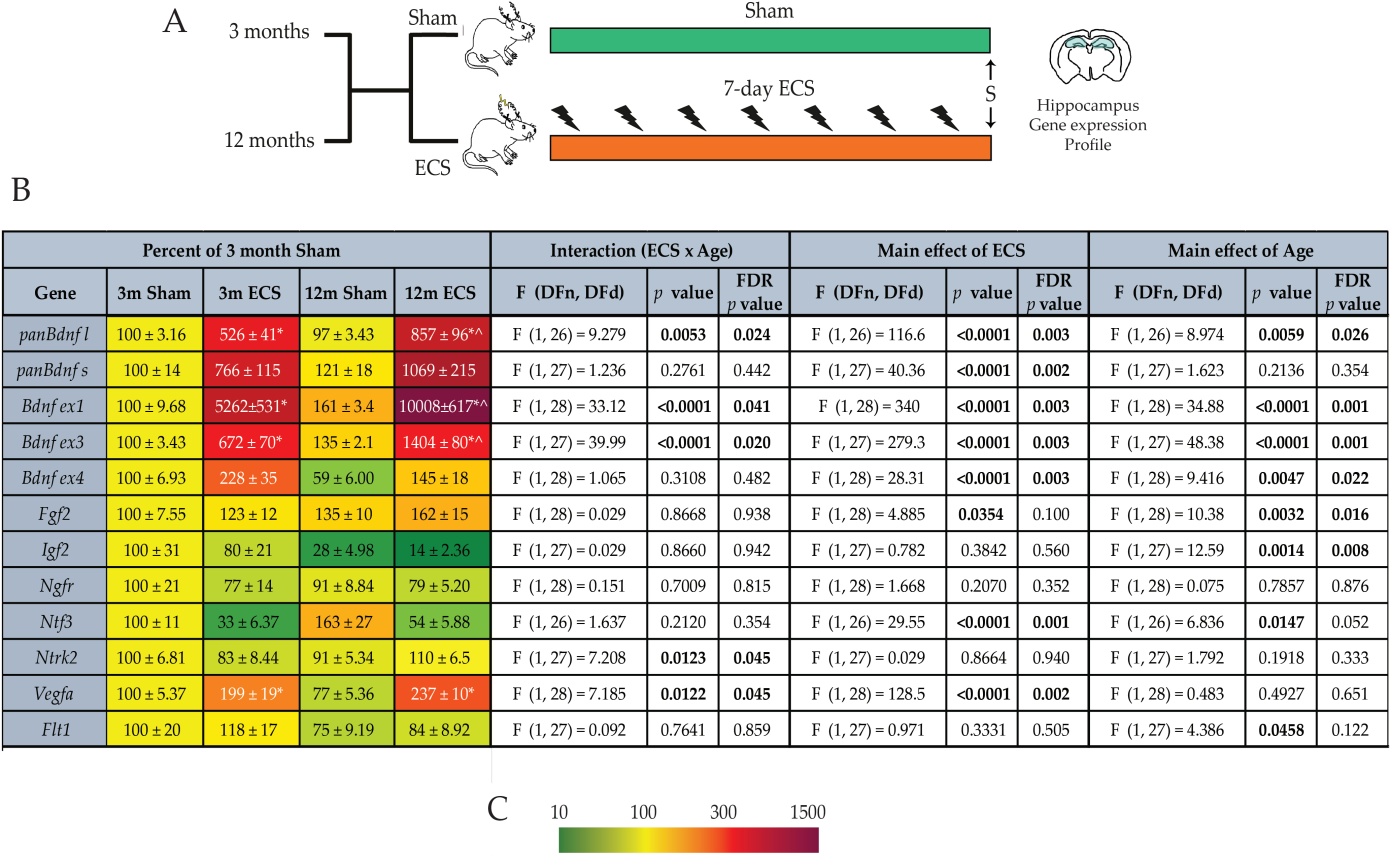
Chronic electroconvulsive seizure (ECS) alters the expression of trophic factors in the hippocampus of young adult and middle-aged rats. (A) Shown is a schematic depicting the experimental paradigm used to test the effects of chronic ECS on the hippocampal expression of trophic factor genes, assessed by quantitative polymerase chain reaction (qPCR), in the hippocampus of young adult and middle-aged rats (for young adult and middle-aged animals: n = 8/group). S denotes the time of killing. (B) Shown are normalized hippocampal gene expression levels for sham- and chronic ECS–treated young adult and middle-aged rats, represented as a percent of young adult sham-treated controls. Heat maps indicate the extent of regulation, with upregulated genes represented in red and downregulated genes represented in green (key, C). Results are expressed as mean ± SEM. Bonferroni post hoc group comparisons were performed when a significant 2-way ANOVA interaction was noted between chronic ECS and age, *P < .05 represents chronic ECS group compared with age-matched sham groups, and ^*P *< .05 middle-aged chronic ECS cohort compared with young adult chronic ECS cohort.

Given the process of aging results in a distinct trophic milieu in the hippocampus (Silhol et al., 2005), we examined whether the impact of chronic ECS on trophic factor expression, which is implicated in the cytoarchitectural, neurogenic, and behavioral actions of chronic ECS (Vaidya et al., 1999; Shirayama et al., 2002; Sairanen et al., 2005; Maynard et al., 2018; Duman et al., 2021), varied based on age (Figure 2A). Although chronic ECS robustly increased the brain derived neurotrophic factor (*Bdnf*) long (*Bdnfl*) and short (*Bdnfs*) 3ʹ UTR variants, we did note a significant interaction of chronic ECS and age for the regulation of the *Bdnfl* variant (Figure 2B; supplementary Figure 2A and B), which was potentiated in the middle-aged cohort. We also examined the regulation of select exon-specific *Bdnf* transcript variants and noted a robust chronic ECS-mediated upregulation of the *Bdnf ex1*, *Bdnf ex3*, and *Bdnf ex4* variants at both ages (Figure 2B; supplementary Figure 2A and B). Significant interaction effects of chronic ECS and age were observed for *Bdnf ex1* and *Bdnf ex3*, with a greater magnitude of upregulation in the middle-aged cohort. We observed a significant chronic ECS and age interaction for vascular endothelial growth factor A (*Vegfa*) mRNA (Figure 2B; supplementary Figure 2A and B), but not for fibroblast growth factor 2 (*Fgf2*), neurotrophin 3 (*Ntf3*), and insulin-like growth factor 2 (*Igf2*) expression. Further, we noted significant main effects of chronic ECS on hippocampal *Fgf2*, *Ntf3*, and *Vegfa* mRNA and of age for *Fgf2*, *Ntf3*, *Igf2*, and the VEGF receptor *Flt1* expression (Figure 2B; supplementary Figure 2A and B). Collectively, these results indicate that chronic ECS exerts robust effects on the hippocampal expression of several trophic factors, with an age-dependent variation in the magnitude and nature of regulation.

### Chronic ECS Differentially Impacts Hippocampal Neurogenesis in Young and Middle-Aged Animals

**Figure 3.**
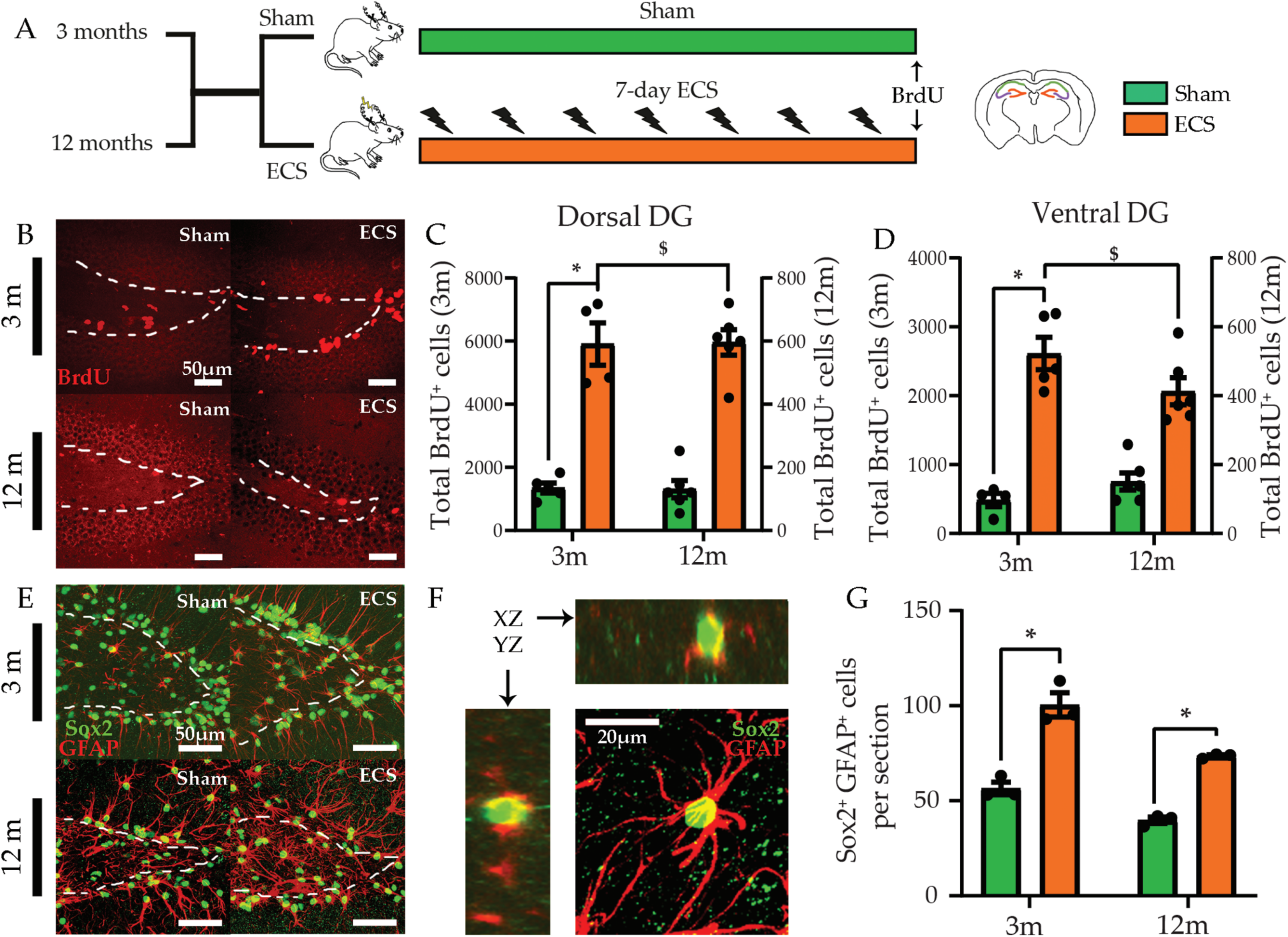
Chronic electroconvulsive seizure (ECS) differentially regulates adult hippocampal neurogenesis in young and middle-aged rats. (A) Shown is a schematic depicting the experimental paradigm used to test the effects of chronic ECS on adult hippocampal neurogenesis in young adult and middle-aged rats. S denotes the time of killing. (B) Shown are representative images of bromodeoxyuridine (BrdU)-positive cells (red) within the hippocampal subgranular zone (SGZ), outlined as a broken white line, of sham- and chronic ECS–treated young adult and middle-aged rats. (C) Chronic ECS led to an increase in total BrdU-positive cells in the dorsal dentate gyrus (DG) of young adult rats but not in the middle-aged cohort. (D) Chronic ECS led to an increase in total BrdU-positive cells in the ventral DG of young adult rats but not in the middle-aged cohort of rats. The y-axes for the number of BrdU-positive cells differ in scale for the young adult and middle-aged cohort. (E) Shown are representative double immunofluorescence images of Sox2^+^ (green) and GFAP^+^ (red) quiescent neural progenitors in the hippocampal SGZ, outlined as a broken white line, of sham- and chronic ECS–treated young adult and middle-aged cohorts. (F) Shown is a representative confocal *z*-stack of Sox2 (green)-GFAP (red) stained quiescent neural progenitors. (G) Chronic ECS led to an increase in the number of Sox2^+^-GFAP^+^ double-positive cells in the dorsal DG of both young adult and middle-aged rats. Results are expressed as mean ± SEM (for young adult and middle-aged experimental cohorts: n = 4–6 per group). Bonferroni post hoc group comparisons were performed when a significant 2-way ANOVA interaction was noted between chronic ECS and age, *P < .05, chronic ECS compared with age-matched sham groups, ^*P *< .05 middle-aged chronic ECS cohort compared with young adult chronic ECS group, and $*P *< .05 young adult chronic ECS cohort compared with middle-aged chronic ECS cohort.

We next assessed whether the influence of chronic ECS on adult hippocampal progenitor turnover varied across young adult and middle-aged cohorts (Figure 3A and B). We noted a significant chronic ECS and age interaction as well as main effects for chronic ECS and age for the number of BrdU⁺ cells within the SGZ in the dorsal and ventral DG (Figure 3C and D; supplementary Table 2). Chronic ECS evoked an age-dependent differential regulation of hippocampal progenitor proliferation, with robust increases restricted to the young adult cohort. Further, we noted significant main effects for chronic ECS and age for Sox2⁺-GFAP⁺ quiescent neural stem cell numbers in the dorsal DG (Figure 3E–G; supplementary Table 2), indicating that although chronic ECS enhanced Sox2⁺-GFAP⁺ quiescent neural stem cell number at both ages examined, the robust enhancement of hippocampal progenitor proliferation was more apparent in the young adult cohort.

### Chronic ECS Evokes an Age-Dependent Reduction in Number of Reelin ^*+*^ Cells

**Figure 4.**
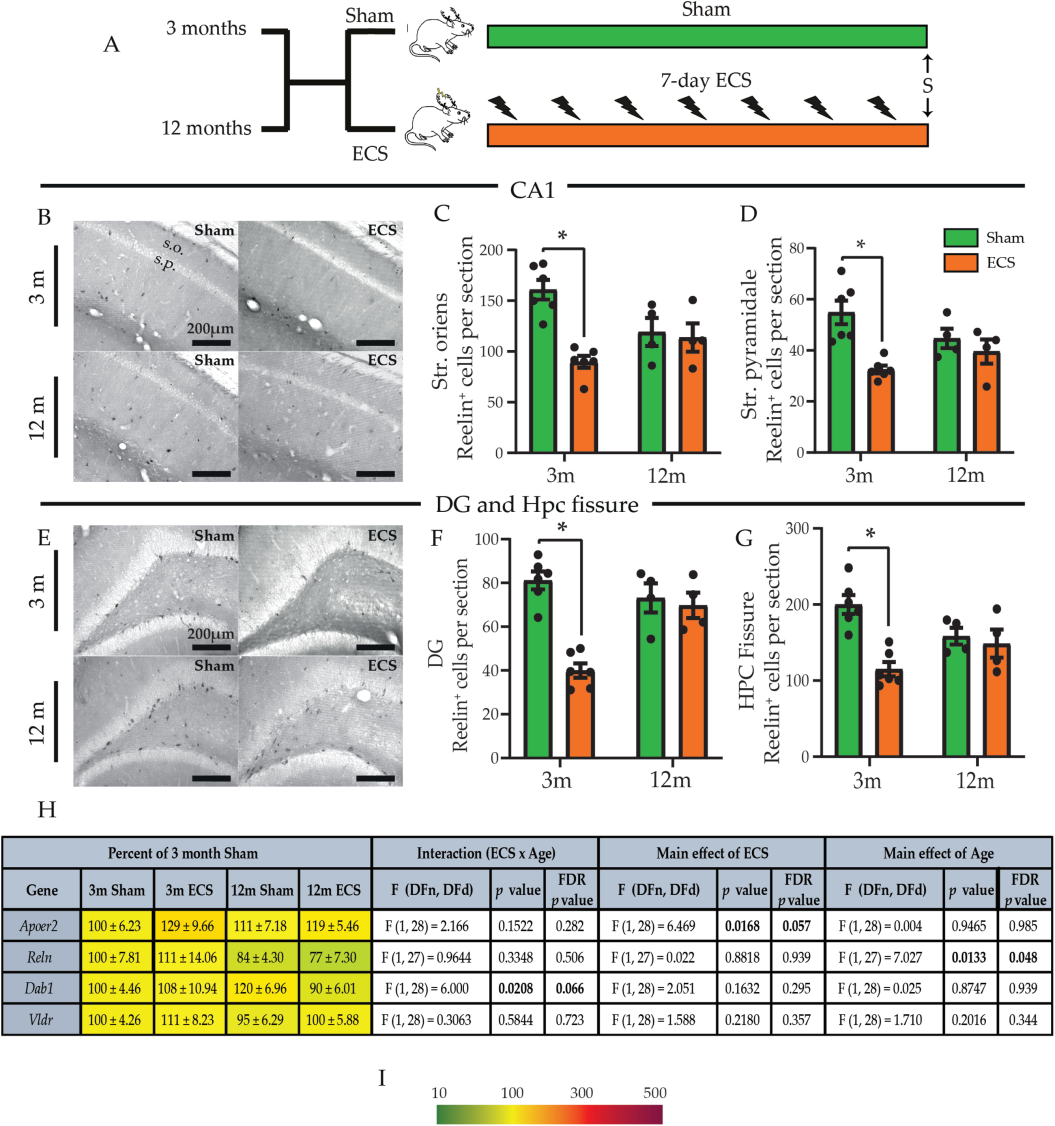
Chronic electroconvulsive seizure (ECS) evokes a reduction in the number of reelin^+^ cells in the hippocampus of young adult but not middle-aged animals. (A) Shown is a schematic depicting the experimental paradigm used to test the effects of chronic ECS on the number of reelin^+^ cells, in the hippocampus assessed by immunohistochemistry, and the expression of reelin pathway signaling genes in the hippocampus of young adult and middle-aged rats. S denotes the time of killing. (B) Shown are representative images of reelin^+^ cells in the hippocampal CA1 subfield of sham- and chronic ECS–treated young adult and middle-aged rats. Chronic ECS led to a decline in the number of reelin^+^ cells in the (C) stratum oriens (s.o.) and the (D) stratum pyramidale (s.p.) layer of the hippocampal CA1 subfield of young adult rats but not in the middle-aged cohort of rats. (E) Shown are representative images of reelin^+^ cells in the hippocampal dentate gyrus (DG) subfield and hippocampal fissure of sham- and chronic ECS–treated young adult and middle-aged rats. Chronic ECS led to a decline in the number of reelin^+^ cells in the (F) hippocampal DG subfield and the (G) hippocampal fissure of the young adult cohort but not in the middle-aged cohort of animals. (H) Shown are normalized hippocampal gene expression levels for Reelin signaling pathway associated genes following chronic ECS in young adult and middle-aged rats, represented as a percentage of the young adult sham-treated controls. Heat maps indicate the extent of regulation, with upregulated genes represented in red and downregulated genes represented in green (key, I). Results are expressed as mean ± SEM (for young adult and middle-aged animals: [1] reelin immunohistochemistry, n = 4–6/group; [2] qPCR, n = 8/group). Bonferroni post hoc group comparisons were performed when a significant 2-way ANOVA interaction was noted between chronic ECS and age, *P < .05, chronic ECS cohort compared with age-matched sham groups.

We next assessed the influence of chronic ECS on the extracellular glycoprotein reelin, which plays a key role in synaptogenesis, dendrite morphology, and progenitor migration in the developing and mature nervous system (Del Río et al., 1997; Pesold et al., 1998; Bosch et al., 2016) (Figure 4A). We noted a significant chronic ECS and age interaction and main effects of chronic ECS on reelin⁺ cell number in the stratum oriens and stratum pyramidale layer of the CA1 subfield, with a significant decline in reelin⁺ cells restricted to young adulthood (Figure 4B–D; supplementary Table 2). No effect of chronic ECS was observed in the CA3 subfield (supplementary Figure 3A–D) at both ages. Cell counting analysis of reelin⁺ cell numbers within the DG subfield (Figure 4E and F) and hippocampal fissure (Figure 4G; supplementary Figure 3E) indicated a significant chronic ECS and age interaction, with a decline in reelin⁺ cell numbers restricted to young adults (Figure 4F and G; supplementary Table 2). Although we did not observe a significant chronic ECS and age interaction effect for reelin⁺ cell numbers in the hilus (supplementary Figure 3F and G), we did note a significant main effect of chronic ECS (supplementary Table 2). We then assessed whether chronic ECS influences the hippocampal expression of reelin signaling pathway components, namely the apolipoprotein E receptor 2 (*Apoer2*) and very low-density lipoprotein receptor (*Vldlr*) that bind reelin, Disabled homolog 1 (*Dab1*), an adaptor protein involved in reelin intracellular signaling, and reelin (*Reln*) (Jossin, 2020) (Figure 4H and I; supplementary Figure 4A and B). We observed a significant interaction effect for *Dab1* mRNA expression and significant main effects of chronic ECS for *Apoer2* and of age for *Reln* mRNA levels (Figure 4H; supplementary Figure 4A and B). Our findings indicate that the effects of chronic ECS on reelin⁺ cells in the hippocampus were highly dependent on age, with robust decreases in reelin⁺ cell number restricted to young adulthood.

### Chronic ECS Regulates Hippocampal ECM-Associated Gene Expression

**Figure 5.**
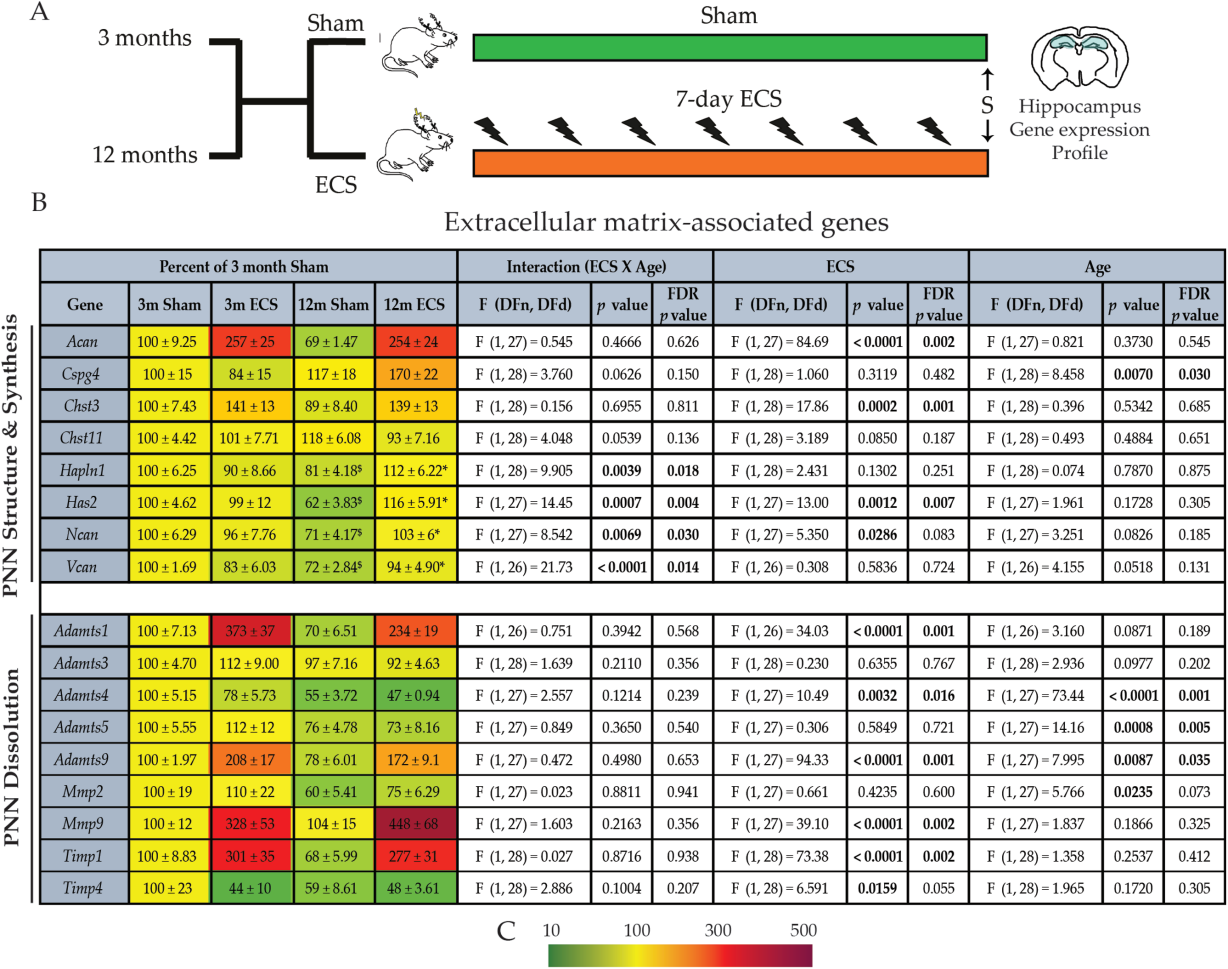
Chronic electroconvulsive seizure (ECS) alters the expression of extracellular matrix–associated genes in the hippocampus of young adult and middle-aged rats. (A) Shown is a schematic depicting the experimental paradigm used to test the effects of chronic ECS on the hippocampal expression of extracellular matrix–associated genes, assessed by quantitative polymerase chain reaction (qPCR), in the hippocampus of young adult and middle-aged rats (for young adult and middle-aged animals: n = 8/group). S denotes the time of killing. (B) Shown are normalized hippocampal gene expression levels for chronic ECS– and sham-treated young adult and middle-aged rats, represented as a percent of young adult sham-treated controls. Heat maps indicate the extent of regulation, with upregulated genes represented in red and downregulated genes represented in green (key, C). Results are expressed as mean ± SEM. Bonferroni post hoc group comparisons were performed when a significant 2-way ANOVA interaction was noted between chronic ECS and age, *P < .05, chronic ECS compared with age-matched sham groups, ^$^*P *< .05 middle-aged sham cohort compared with young adult sham cohort, and ^*P *< .05 young adult chronic ECS cohort compared with middle-aged chronic ECS cohort.

Chronic ECS is reported to alter gene expression and activity of enzymatic machinery that regulates the ECM, namely matrix metallopeptidase (MMP2 and MMP9) and endogenous modulators of these enzymes (Newton et al., 2003a; Benekareddy et al., 2008; Alaiyed and Conant 2019). Further, rapid action antidepressants such as ketamine target ECM structures such as the PNNs that are preferentially deposited around PV positive (PV^+^) interneurons (Venturino et al., 2021). We observed a significant chronic ECS and age interaction for the gene expression of PNN components, Hyaluronan and proteoglycan link protein 1 (*Hapln1*), Neurocan (*Ncan*), Versican (*Vcan*), and the PNN synthesizing enzyme, Hyaluronan synthase 2 (*Has2*) (Figure 5B; supplementary Figure 5A and B), with *Hapln1*, *Has2*, *Ncan*, and *Vcan* mRNA significantly increased only in the middle-aged cohort. We observed significant main effects for chronic ECS on PNN components and synthesizing machinery: Aggrecan (*Acan*), Carbohydrate sulfotransferase 3 (*Chst3*), *Has2*, and *Ncan*, and PNN dissolution enzymes: a disintegrin and metalloproteinase with thrombospondin motifs (*Adamts1*, *Adamts4*, *Adamts9*), matrix metalloproteinase (*Mmp9*), and tissue inhibitor of metalloproteinases (*Timp1* and *Timp4*) (Figure 5B; supplementary Figure 5A and B). A significant main effect of age was observed for Chondroitin sulfate proteoglycan 4 (*Cspg4*), *Adamts4*, *Adamts5*, *Adamts9*, and *Mmp2* (Figure 5B; supplementary Figure 5A and B). These findings indicate that chronic ECS modulates the expression of several genes associated with PNN formation and breakdown in both an age-dependent and independent pattern.

### Chronic ECS Regulates Perineuronal Net Expression Within Hippocampal Subfields

**Figure 6.**
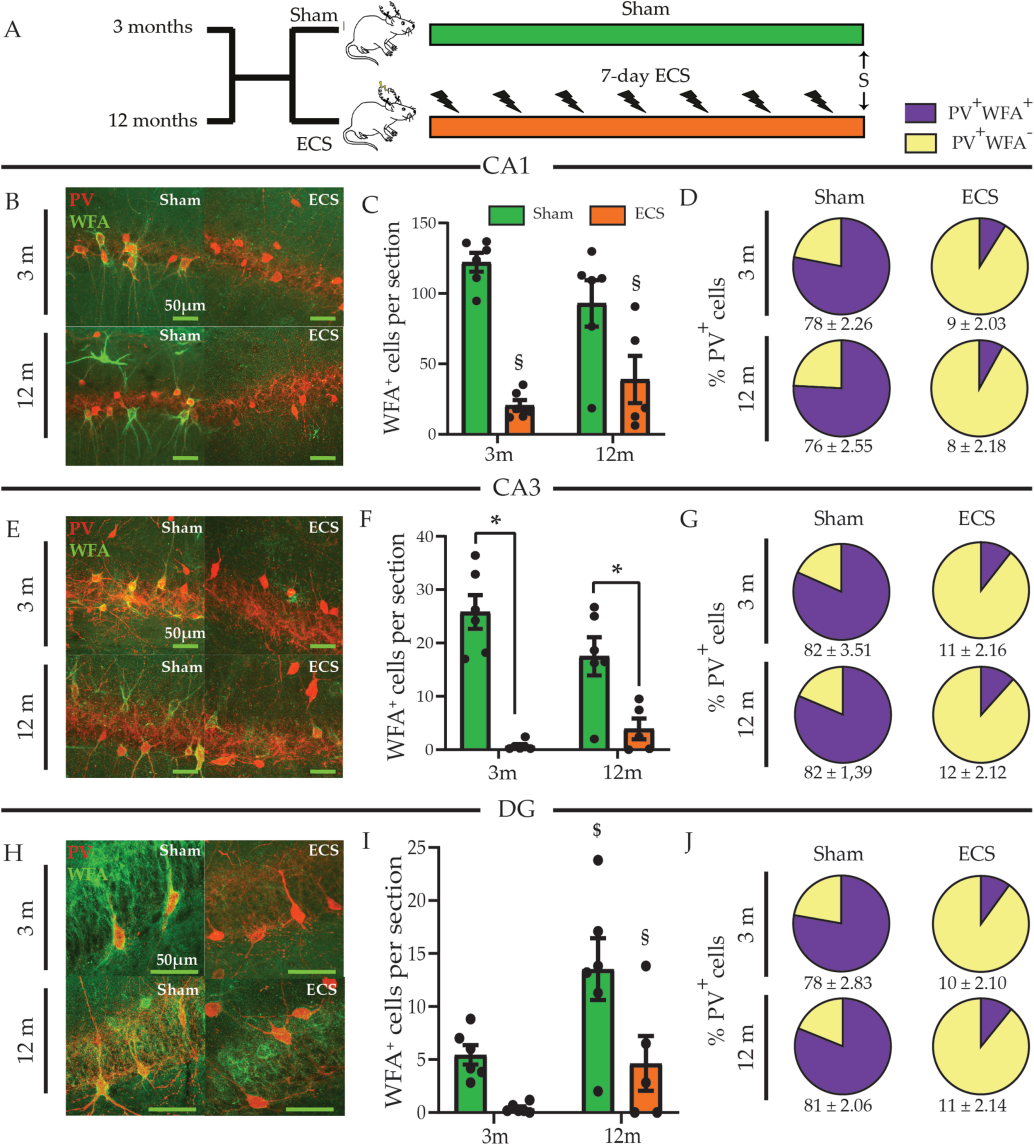
Chronic electroconvulsive seizure (ECS) results in a reduction in the number of perineuronal nets in the hippocampus of young adult and middle-aged rats. (A) Shown is a schematic depicting the experimental paradigm used to test the effects of chronic ECS on the extracellular matrix structure of the perineuronal nets (PNNs), assessed by immunohistochemistry for the plant lectin Wisteria Floribunda Agglutinin (WFA) in the hippocampus of young adult and middle-aged rats. S denotes the time of killing. (B) Shown are representative double immunofluorescence images of WFA^+^ PNNs (green) and parvalbumin (PV)^+^ (red) neurons in the hippocampal CA1 subfield of sham- and chronic ECS–treated young adult and middle-aged cohorts. Chronic ECS led to a stark decline in (C) the number of WFA-stained PNNs and (D) the percentage of PV^+^ cells surrounded by WFA-stained PNNs in the hippocampal CA1 subfield of both young adult and middle-aged rats. (E) Shown are representative double immunofluorescence images of WFA^+^ PNNs (green) and PV^+^ (red) neurons in the hippocampal CA3 subfield of sham- and chronic ECS–treated young adult and middle-aged rats. Chronic ECS lead to a reduction in (F) the number of WFA^+^ PNNs and (G) the percentage of PV^+^ cells surrounded by WFA-stained PNNs in the hippocampal CA3 subfield of both young adult and middle-aged cohorts. (H) Shown are representative double immunofluorescence images of WFA^+^ PNNs (green) and PV^+^ (red) neurons in the hippocampal dentate gyrus (DG) subfield of sham- and chronic ECS–treated young adult and middle-aged rats. Chronic ECS led to a decline in (I) the number of WFA^+^ PNNs and (J) the percentage of PV^+^ cells surrounded by WFA^+^ PNNs in the hippocampal DG subfield of middle-aged but not young adult animals. Results are expressed as mean ± SEM (for young adult and middle-aged animals: n = 4–6/group). Bonferroni post hoc group comparisons were performed when a significant 2-way ANOVA interaction was noted between chronic ECS and age, *P < .05, chronic ECS compared with age-matched sham groups. ^$^ denotes significant main effect of chronic ECS, ^§^ denotes significant main effect of age.

We next assessed whether chronic ECS influences hippocampal PNN numbers in an age-dependent manner (Figure 6A). We noted a significant chronic ECS and age interaction for WFA⁺-PNN numbers in the CA3 (Figure 6E and F; supplementary Table 2) but not in the CA1 and DG (Figure 6B, C, H and I; supplementary Table 2) hippocampal subfields. Post-hoc group comparisons indicated a significant reduction in WFA⁺-PNN numbers in the CA3 subfield of both young adult and middle-aged rats (Figure 6F; supplementary Table 2). We also noted significant main effects of chronic ECS (supplementary Table 2) for the CA1 (Figure 6C), CA3 (Figure 6F), and DG (Figure 6I) hippocampal subfields. Further, we observed a significant main effect of age only in the DG (Figure 6I; supplementary Table 2).

PNNs are differentially distributed across distinct hippocampal subfields (supplementary Figure 6A) and predominantly deposited around PV⁺-interneurons, although their presence has also been noted around other cell types (Giamanco and Matthews 2012; Sorg et al., 2016). We addressed whether the proportion of PV⁺ interneurons surrounded by WFA⁺-PNNs was altered by chronic ECS at both ages. Chronic ECS resulted in the robust dissolution of PNNs surrounding PV⁺ interneurons in the CA1 (Figure 6D; supplementary Figure 6B and C), CA3 (Figure 6G; supplementary Figure 6D), and DG (Figure 6J; supplementary Figure 6E) hippocampal subfields at both ages. We noted significant main effects of chronic ECS (supplementary Table 2) for the percent colocalization of WFA with PV in the CA1 (Figure 6D; supplementary Figure 6C), CA3 (Figure 6G; supplementary Figure 6D), and DG (Figure 6J; supplementary Figure 6E) subfields. These results indicate that chronic ECS results in almost complete dissolution of PNNs surrounding PV^+^ interneurons in the hippocampi of both young adult and middle-aged animals.

## DISCUSSION

Here, we provide novel evidence that although chronic ECS modulates despair-like behavior in young adult and middle-aged male rats to a similar extent, these behavioral effects are accompanied by unique and overlapping changes in the regulation of trophic factors, ECM-associated genes, as well as changes in adult hippocampal neurogenesis, reelin expression, and PNNs. These findings indicate that age is a critical variable in determining the chronic ECS-evoked molecular and cellular changes, raising the intriguing possibility of mechanistic distinctions that may drive therapeutic responses to ECT at different ages.

Pharmacological antidepressants can evoke differential effects on despair-like and cognitive behavior based on age at onset of treatment (Yau et al., 2002; Herrera-Pérez et al., 2010; Li et al., 2015; Olivares-Nazario et al., 2016; Fernández-Guasti et al., 2017), with evidence of attenuated behavioral responses in middle-aged and senescent animals. In comparison, we did not observe any significant differences in the chronic ECS evoked a decline in despair-like behavior on the FST between young adult and middle-aged male rats. Prior evidence indicates that chronic ECS enhances total locomotion in exploratory tasks such as the open field test (Li et al., 2007). Given that alterations in total mobility time on the FST are used to interpret changes in despair-like behavior, a caveat to keep in mind is that the potential effects of chronic ECS on total locomotion may also impact behavioral measures on the FST. One of the limitations of our study is that our behavioral analysis is limited to the FST, which is dependent on measures linked to locomotor behavior. A useful addition to address the impact of chronic ECS on antidepressant-like behavior at the 2 ages studied would be behavioral tasks that are not as heavily dependent on locomotor behavior, such as the sucrose consumption test and the novelty suppressed feeding test.

The pattern and magnitude of chronic ECS-evoked regulation of activity-dependent gene (*Arc*, *Egr2*, *Egr3*, *Fos*) expression also did not vary substantially, suggesting that the impact of ECS on these neuronal depolarization responsive gene loci appears to be predominantly age independent. We observed that the magnitude of chronic ECS-mediated regulation of hippocampal neurotrophic factor gene expression varied in a gene- and age-dependent manner. Prior studies report that chronic ECS enhances expression of *Bdnf*, *Vegfa*, and *Fgf2* in the hippocampi of young adult animals (Rocamora et al., 1992; Nibuya et al., 1995; Smith et al., 1995; Newton et al., 2003; Altar et al., 2004a). Our results indicate that despite an aging hippocampal milieu, chronic ECS evokes robust increases in *Bdnf*, *Vegfa*, and *Fgf2* mRNA expression, and, intriguingly, the magnitude of regulation is significantly potentiated in the case of the *Bdnf* transcript variants. The greater magnitude of the chronic ECS-evoked transcriptional regulation of specific *Bdnf* transcript variants (*pan Bdnfl*, *Bdnf ex1*, *Bdnf ex3*) in the middle-aged cohort suggests the speculative possibility of a differential recruitment of signaling pathways and epigenetic modifications that drive these age-dependent transcriptional consequences. These findings motivate future experiments to carefully examine the age dependence of the impact of chronic ECS at the *Bdnf* gene locus, given that these could directly impinge on *Bdnf* mRNA stability, trafficking, and local translation, thus impacting the effects of BDNF on structural and functional plasticity in the hippocampus.

Chronic ECS is reported to robustly increase hippocampal progenitor cell proliferation and QNP numbers in young adult animals (Scott et al., 2000; Segi-Nishida et al., 2008; Rotheneichner et al., 2014), which is implicated in mediating the antidepressant-like behavioral effects. Hippocampal neurogenesis is significantly attenuated with aging (Kuhn et al., 1996; Couillard-Despres et al., 2006, 2009; Villeda et al., 2011), and quiescence-associated changes in stem cells are already well established in middle-aged life (Encinas and Sierra 2012). Our data agree with prior reports that middle-aged animals exhibit a steep decline in BrdU^+^ proliferating hippocampal progenitors despite no substantial difference in QNP numbers, suggesting a shift to quiescence and reduced proliferative capacity in the middle-aged neurogenic niche. We find that the chronic ECS-evoked increase in BrdU^+^ hippocampal progenitors is significantly attenuated in the middle-aged cohort (Rotheneichner et al., 2014); however, the scale of increase in QNP numbers evoked by chronic ECS is comparable between the ages. These results indicate that although chronic ECS does drive a similar scale of increase in the QNP pool at both ages, this does not translate into similar numbers of proliferating hippocampal progenitors. This raises the intriguing possibility that unlike the young adulthood window where neurogenic changes are strongly implicated in driving antidepressant-like behavioral changes (Santarelli et al., 2003), it is possible that adult hippocampal neurogenesis may not play as key a role in contributing to the behavioral effects of chronic ECS in the middle-aged cohort. Given concerns of substantial chronic ECS-evoked mortality in aged animals (20–24 months and older), we restricted our studies to a middle-aged cohort with minimal mortality. However, while interpreting the results of our study it is important to keep in mind that the impact of chronic ECS in the middle-aged cohort may not necessarily recapitulate chronic ECS-evoked effects in a much older cohort, with substantially curtailed possibilities of plasticity.

Our observations of neurogenic, neurotrophic, and activity-dependent gene expression changes evoked by chronic ECS in both the young adult and middle-aged cohort raises the intriguing possibility that this treatment modality may potentially recapitulate the molecular and cellular hallmarks of a “critical period plasticity-like state” (Umemori et al., 2018). To address this hypothesis, we examined the influence of chronic ECS on the expression and deposition of ECM components that shift dynamically across critical periods, including the secreted glycoprotein reelin, which regulates dendritogenesis and synaptogenesis (Jossin, 2020; Faini et al., 2021). Strikingly, we noted an age-dependent downregulation of reelin^+^ cell numbers in multiple hippocampal subfields observed in the young adult but not the middle-aged cohort following chronic ECS. Reelin function is implicated in the maintenance of mature hippocampal neuronal dendritic and synaptic architecture, with evidence suggesting that an attenuation of reelin signaling in mature networks results in the reactivation of robust dendritogenesis, a feature normally associated with developing neuronal networks (Ampuero et al., 2017). Studies hypothesize that reelin signaling while driving dendritogenesis in immature neurons may contribute to the stability of established dendritic architecture in mature neurons, raising the speculative possibility that the reduction of reelin expression following chronic ECS would serve to favor a milieu that facilitates dendritic reorganization. It is rather intriguing that the influence of chronic ECS on reelin expression was restricted to the young adulthood window, suggesting that the impact of chronic ECS on this secreted glycoprotein that can regulate dendritogenesis is highly age dependent.

A hallmark cellular feature associated with the closure of critical periods is the deposition of PNNs (Pizzorusso et al., 2002), which are lattice-like, proteoglycan-rich, ECM structures that coalesce around specific neuronal populations, in particular PV^+^ inhibitory interneurons, and are linked to the limiting of neuronal plasticity in mature neuronal networks (Bozzelli et al., 2018; Sigal et al., 2019; Willis et al., 2022). Prior evidence indicates that pharmacological antidepressants reopen “critical period-like plasticity” in adulthood, likely via the recruitment of Tropomyosin receptor kinase B (TrkB) signaling in PV^+^ interneurons (Maya Vetencourt et al., 2008; Guirado et al., 2014; Lesnikova et al., 2021; Mukhopadhyay et al., 2021). Diverse pharmacological antidepressants, including fluoxetine (Maya Vetencourt et al., 2008), venlafaxine (Alaiyed et al., 2019), and ketamine (Venturino et al., 2021), are reported to impact PNN integrity likely via the upregulation of PNN dissolution–associated enzymatic machinery, namely the MMPs, creating a milieu that promotes synaptic plasticity, synaptogenesis, and reorganization of network properties (Alaiyed and Conant, 2019). To the best of our knowledge, the impact of chronic ECS on PNN modulation has not been addressed, despite several reports indicating robust effects of chronic ECS on both the expression and enzymatic activity of specific MMPs (Newton et al., 2003; Benekareddy et al., 2008; Girgenti et al., 2011), implicated in PNN dissolution and synaptic plasticity. Here, we show that chronic ECS results in the transcriptional regulation of multiple genes associated with PNN formation and breakdown exhibiting both an age-dependent and independent pattern. Further, our results indicate a predominantly age-invariant effect of chronic ECS on PNNs in multiple hippocampal subfields, with a robust reduction in PNN numbers in the CA1, CA3, and DG at both ages examined, as well as evidence of the significant disruption of PNNs surrounding PV⁺ interneurons in all these hippocampal subfields at both ages. Although we observed reduced labeling by WFA, a plant lectin that selectively binds to CSPGs (Härtig et al., 1992), we cannot comment on the impact of chronic ECS on the protein expression of selective PNN components, which may also be impacted by chronic ECS. It is likely that the enhanced expression of ADAMTS (*Adamts1*, *Adamts9*) and MMPs (*Mmp9*), as well as prior evidence clearly indicating enhanced proteolytic activity following chronic ECS (Benekareddy et al., 2008), may contribute to the breakdown of PNNs. It is important to note that transcriptional changes in PNN synthesis and PNN dissolution–associated genes may not necessarily recapitulate changes in PNN numbers within the hippocampus. This point should be kept in mind while interpreting chronic ECS-evoked transcriptional changes in PNN-linked machinery. Dissolution of PNNs may reopen a “critical-period” plasticity-like window that could facilitate a milieu for heightened dendritogenesis, axonal sprouting, and synaptic reorganization, which arise to a differential magnitude in response to both pharmacological antidepressants and rapid-acting antidepressant modalities such as ketamine and chronic ECS and are implicated in driving therapeutic responses (Castrén and Hen, 2013; Sorg et al., 2016; Umemori et al., 2018b). In this regard, it is intriguing that prior reports suggest a role for PNN dissolution in mood-related behavior, with chondroitinase ABC treatment in the rat ventral hippocampus associated with antidepressant-like behavioral responses on the FST (Donegan and Lodge, 2017). One can speculate that the dissolution of PNNs, in particular those encapsulating PV⁺ interneurons, following chronic ECS in both young adults and middle-aged animals could exert robust effects on projection neurons in the hippocampus, modulating rhythmogenesis in this key limbic network and thus influencing mood-related behaviors (Alaiyed et al., 2019).

Collectively, our results indicate that although chronic ECS evokes overlapping changes in activity-dependent and ECM-related gene expression and the dissolution of PNNs in hippocampal subfields, there are unique age-dependent differences in the nature and magnitude of transcriptional regulation of trophic factors, regulation of hippocampal neurogenesis, and reelin expression. This raises the intriguing possibility that chronic ECS may recruit distinct mechanisms to drive the robust antidepressant-like behavioral changes in the middle-aged cohort compared with those targeted in young adult animals. Our studies were performed in stress-naïve animals, similar to the preponderance of chronic ECS literature that has explored effects in naïve animals (Duman and Vaidya, 1998). This raises the intriguing question of whether the effects of chronic ECS, or the molecular and cellular mechanisms targeted by this treatment modality, would vary based on a history of chronic stress or in animal models of depression. Our findings motivate future investigation to identify the molecular, cellular, and circuit-specific changes that mechanistically contribute to the therapeutic effects of rapid-acting antidepressant treatment modalities like chronic ECS and underscore the importance of considering age as a critical variable when addressing the mechanisms targeted by chronic ECS.

## Supplementary Materials

Supplementary data are available at *International Journal of Neuropsychopharmacology (IJNPPY)* online.

pyad008_suppl_Supplementary_Figures

pyad008_suppl_Supplementary_Table_S1

pyad008_suppl_Supplementary_Table_S2

pyad008_suppl_Supplementary_Table_S3

## Data Availability

The data underlying this article will be shared on request to the corresponding author.

## Notes

This work was supported by intramural support to V.A.V. from the Tata Institute of Fundamental Research and Department of Atomic Energy, Mumbai (grant reference no. RTI4003) and from the Department of Biotechnology, Government of India (grant no. BT/COE/34/SP17426/2016). We acknowledge support to R.S.D. from National Institute of Mental Health grant MH045481.

## Interest Statement

Brendan Hare is currently employed by Click Therapeutics. Other authors have no conflict of interest to declare.
