# Supplementary material for: Influence of Chronic Electroconvulsive Seizures on Plasticity-Associated Gene Expression and Perineuronal Nets Within the Hippocampi of Young Adult and Middle-Aged Sprague-Dawley Rats
Source: Int J Neuropsychopharmacol. 2023 Mar 4;26(4):294–306. doi: 10.1093/ijnp/pyad008 (PMC10109107; doi:10.1093/ijnp/pyad008)

*Supplementary Figure 1*

*Chronic ECS alters the expression of immediate early genes and reelin-associated genes in the hippocampi of young adult and middle-aged rats.*

1. Shown is a schematic depicting the experimental paradigm used to examine the effects of chronic ECS on the hippocampal expression of immediate early genes and reelin-associated genes, assessed by quantitative polymerase chain reaction (qPCR), in the hippocampi of young adult and middle-aged rats (For young adult and middle-aged animals: n = 8/group). S denotes the time of sacrifice. *(B)* Shown are normalized hippocampal gene expression levels of immediate early genes for sham and chronic ECS treated young adult and middle-aged rats, represented as a percent of young adult sham-treated controls. Results are expressed as mean ± SEM; Bonferroni post hoc group comparisons were performed when a significant two-way ANOVA interaction was noted between chronic ECS and age, **p* < .05 as compared to age-matched sham treated controls, and ^*p* < .05 as compared to the young adult chronic ECS treated cohort.


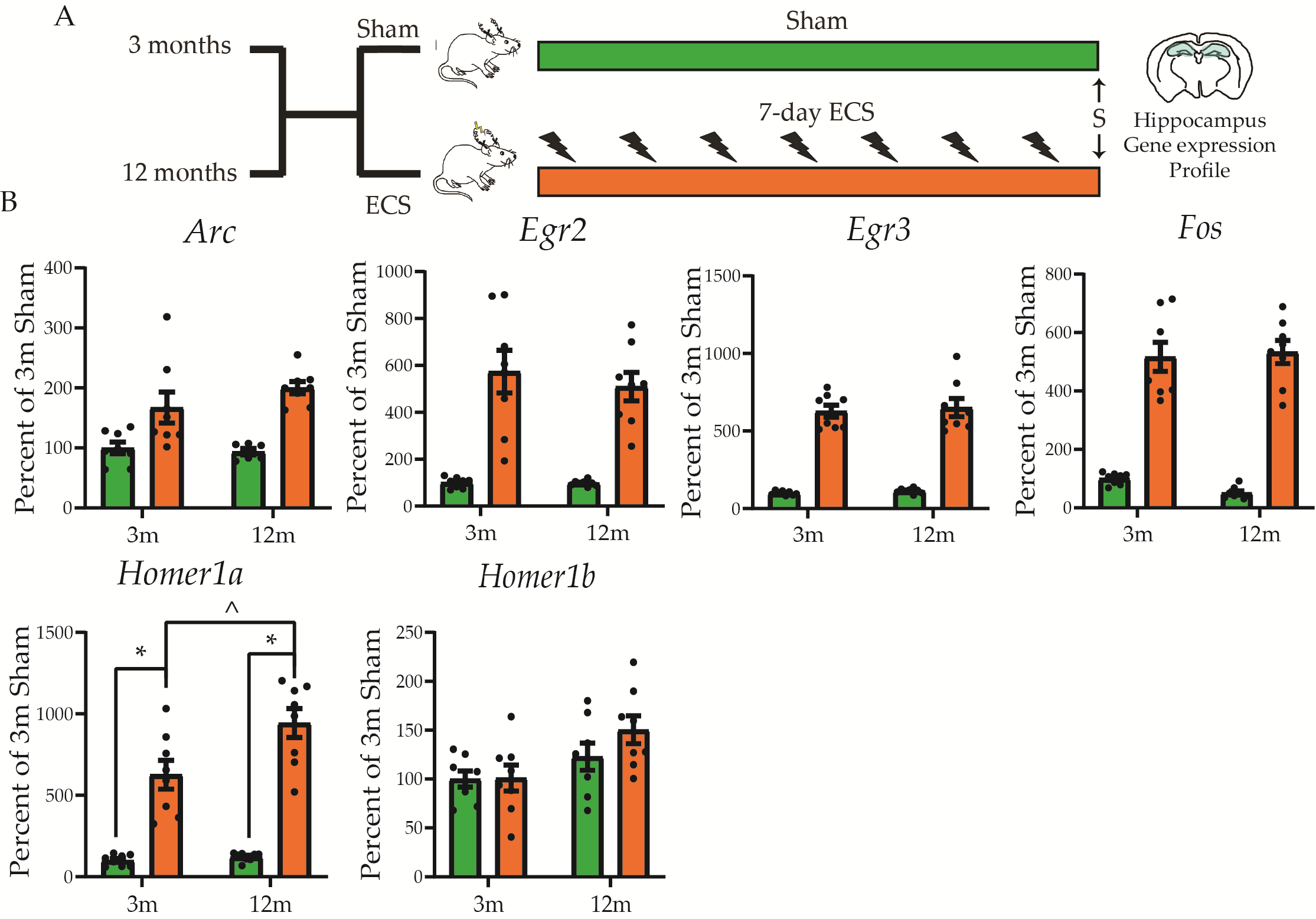


*Supplementary Figure 2*

*Chronic ECS alters the expression of trophic factor genes in the hippocampi of young adult and middle-aged rats.*

1. Shown is a schematic depicting the experimental paradigm used to examine the effects of chronic ECS on the hippocampal expression of trophic factor genes assessed by quantitative polymerase chain reaction (qPCR), in the hippocampi of young adult and middle-aged rats (For young adult and middle-aged animals: n = 8/group). S denotes the time of sacrifice. *(B)* Shown are normalized hippocampal gene expression levels of trophic factor genes for sham and chronic ECS treated young adult and middle-aged rats, represented as a percent of young adult sham-treated controls. Results are expressed as the mean ± SEM; Bonferroni post hoc group comparisons were performed when a significant two-way ANOVA interaction was noted between chronic ECS and age, **p* < .05 as compared to age-matched sham groups, and ^*p* < .05 as compared to the young adult chronic ECS treated cohort.


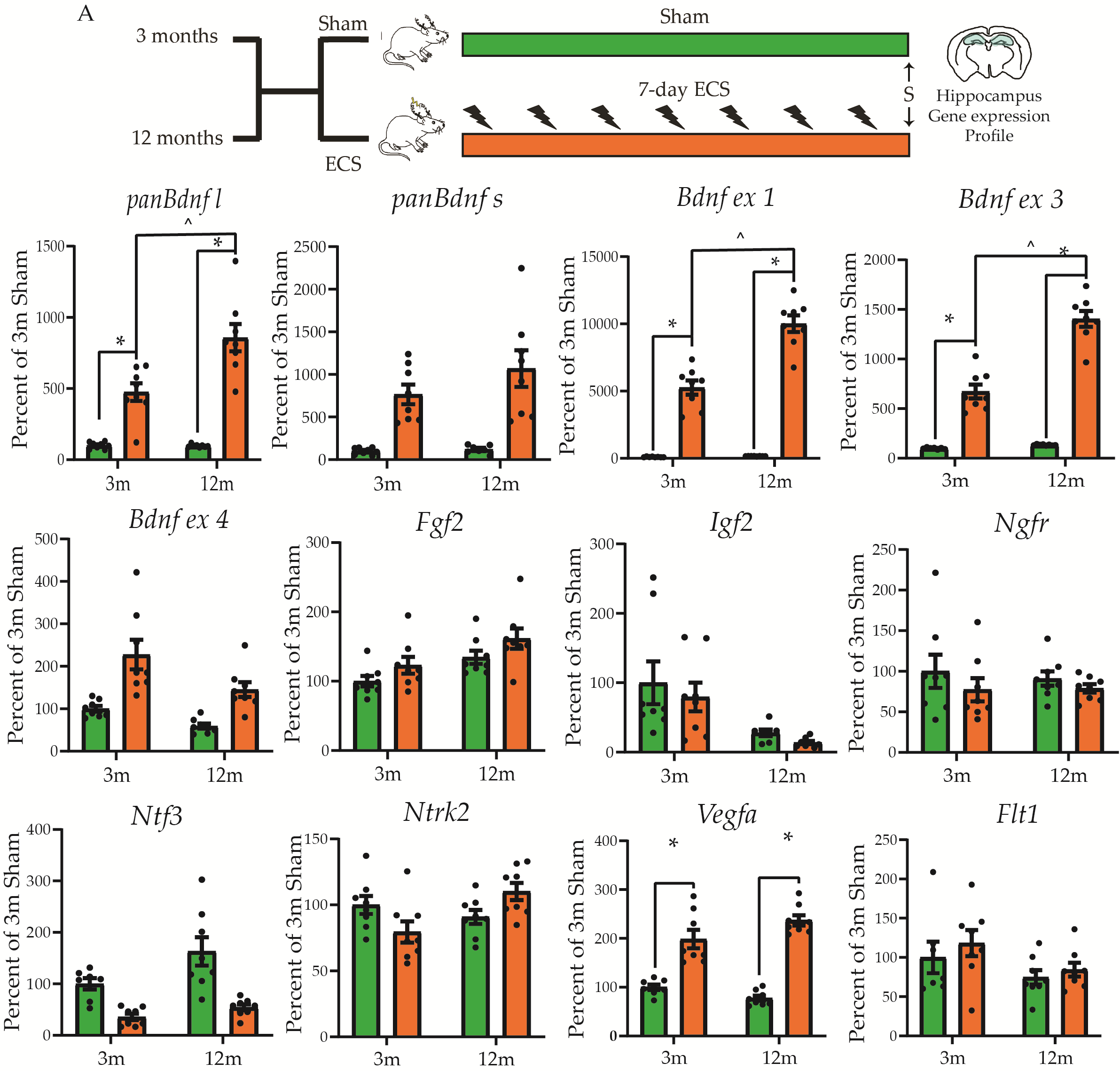


*Supplementary Figure 3*

*Chronic ECS alters the number of Reelin*^+^ *cells in the hippocampus in an age-dependent manner.*

1. Shown is a schematic depicting the experimental paradigm used to examine the effects of chronic ECS on the number of reelin-positive cells, assessed by immunohistochemistry in the hippocampi of young adult and middle-aged rats. S denotes the time of sacrifice. *(B)* Shown are representative images of reelin^+^ cells in the hippocampal CA3 subfield of sham and chronic ECS treated young adult and middle-aged rats. Chronic ECS treatment did not change the number of Reelin^+^ cells in the *(C)* stratum oriens (s.o.) and the *(D)* stratum pyramidale (s.p.) layer of the hippocampal CA3 subfield of young adult rats or middle-aged rats. *(E)* Shown are representative images of Reelin^+^ cells in the hippocampal fissure of sham and chronic ECS treated young adult and middle-aged animals. *(F)* Shown are representative images of Reelin^+^ cells in the hilar region of hippocampus from sham and chronic ECS treated young adult and middle-aged cohorts. *(G)* Chronic ECS did not influence the number of Reelin^+^ cell numbers in the hilus region in the young adult and middle-aged cohort of animals compared to their respective sham treated controls. Results are expressed as mean ± SEM.


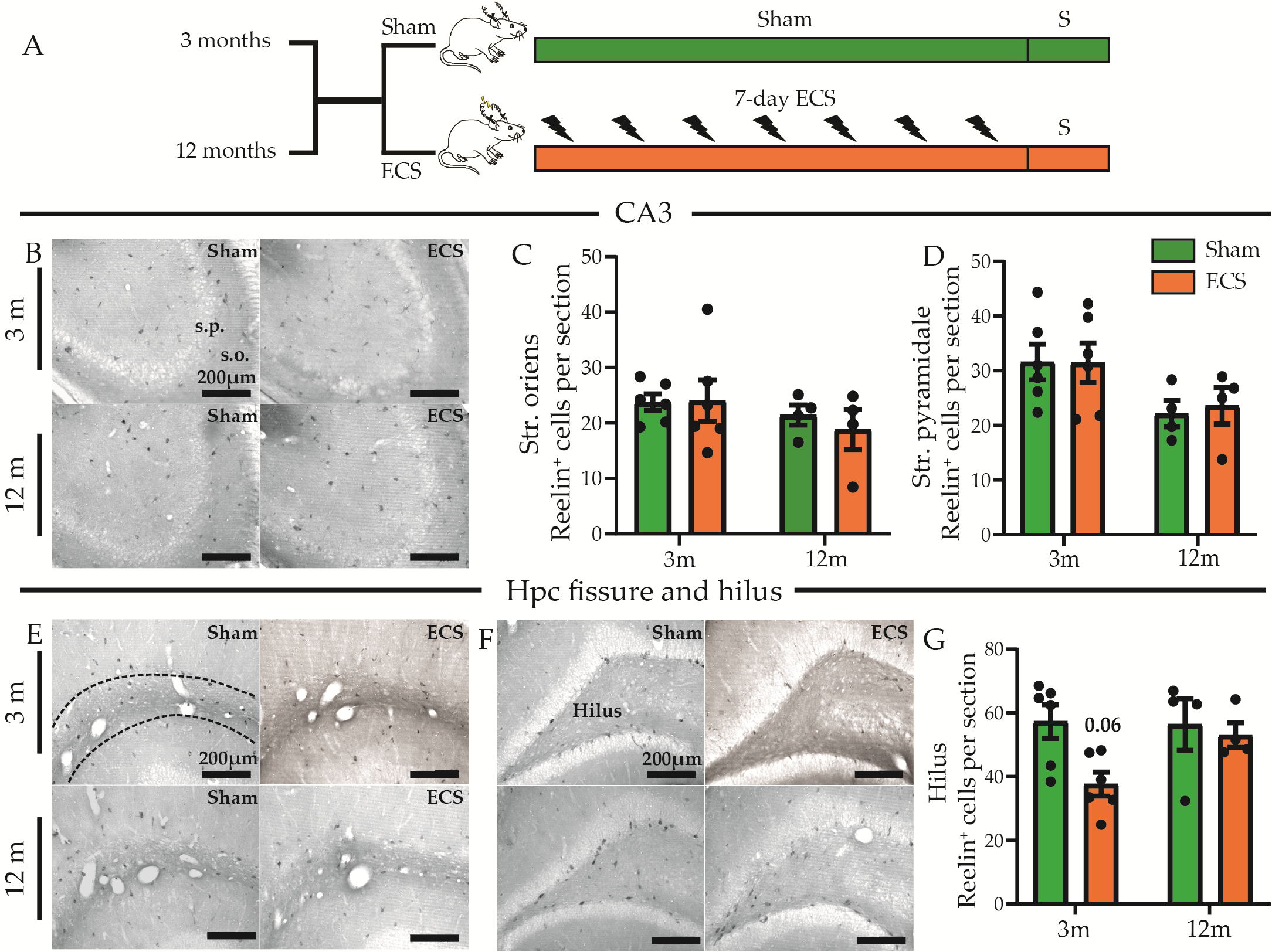


*Supplementary Figure 4*

*Chronic ECS alters the expression of Reelin pathway associated genes in the hippocampi of young adult and middle-aged rats.*

1. Shown is a schematic depicting the experimental paradigm used to examine the effects of chronic ECS on the hippocampal expression of reelin pathway associated genes assessed by quantitative polymerase chain reaction (qPCR), in the hippocampi of young adult and middle-aged rats (For young adult and middle-aged animals: n = 8/group). S denotes the time of sacrifice. *(B)* Shown are normalized hippocampal gene expression levels of reelin associated genes for sham and chronic ECS treated young adult and middle-aged rats, represented as a percent of young adult sham-treated controls. Results are expressed as mean ± SEM;


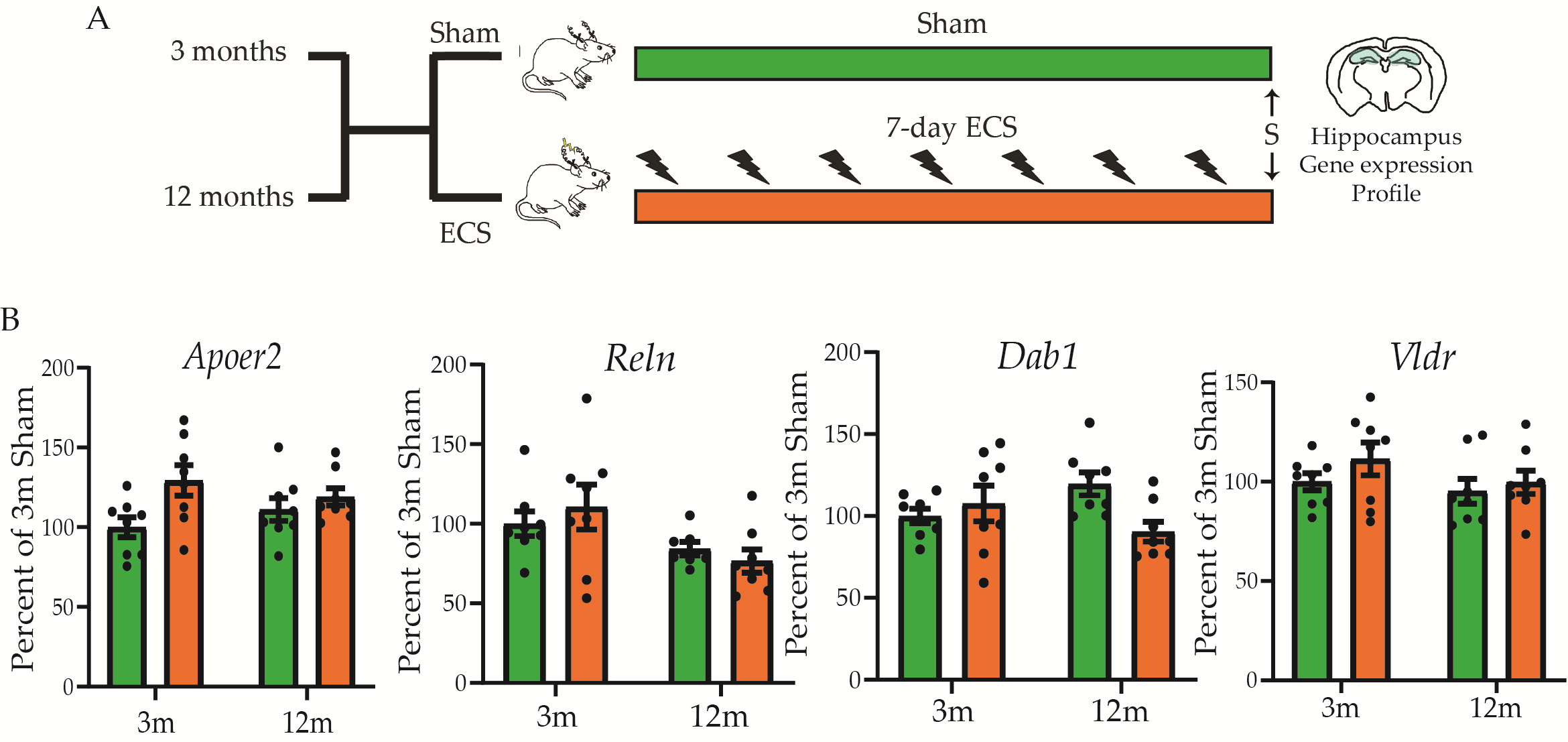


*Supplementary Figure 5*

*Chronic ECS alters the expression of extracellular matrix-associated genes in the hippocampus of young adult and middle-aged rats.*

1. Shown is a schematic depicting the experimental paradigm used to examine the effects of chronic ECS on the hippocampal expression of trophic factor genes assessed by quantitative polymerase chain reaction (qPCR), in the hippocampi of young adult and middle-aged rats (For young adult and middle-aged animals: n = 8/group). S denotes the time of sacrifice. *(B)* Shown are normalized hippocampal gene expression levels of extracellular matrix-associated genes from sham and chronic ECS treated young adult and middle-aged rats, represented as a percent of young adult sham-treated controls. Results are expressed as mean ± SEM; Bonferroni post hoc group comparisons were performed when a significant two-way ANOVA interaction was noted between chronic ECS and age, **p* < .05 as compared to age-matched sham treated groups, ^$^*p* < .05 as compared to young adult sham treated cohort and ^*p* < .05 as compared to young adult chronic ECS cohort.


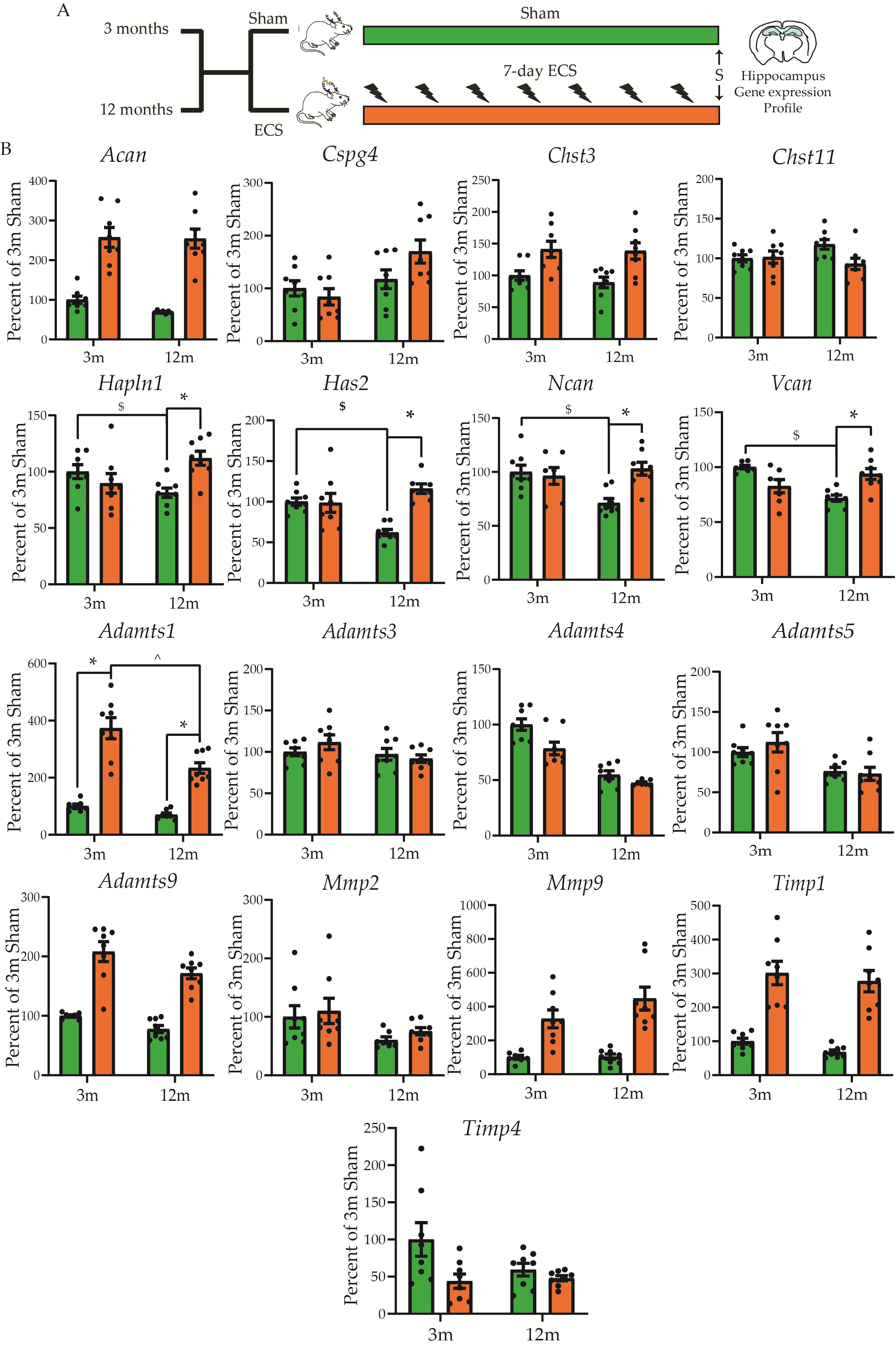


*Supplementary Figure 6*

*Chronic ECS results in a reduction in the number of perineuronal nets in the hippocampus of young adult and middle-aged rats.*

*(A)* Shown is a representative tiled image of WFA^+^ PNNs in the entire hippocampus of a sham treated young adult Sprague-Dawley rat. Subregions of the hippocampus have been marked for clarity and distinct extracellular matrix structures can be seen in the CA1, CA3 and DG hippocampal subfields. *(B)* Shown is a schematic depicting the experimental paradigm used to examine the effects of chronic ECS on the extracellular matrix structure of the perineuronal nets (PNNs), assessed by immunohistochemistry. Chronic ECS lead to a stark decline in the percentage of PV^+^ cells surrounded by WFA-stained PNNs in the hippocampal CA1 subfield (C), in the CA3 subfield (D), and in the DG (F) of both young adult and middle-aged rats. Results are expressed as mean ± SEM (For young adult and middle-aged animals: n = 4-6/group).


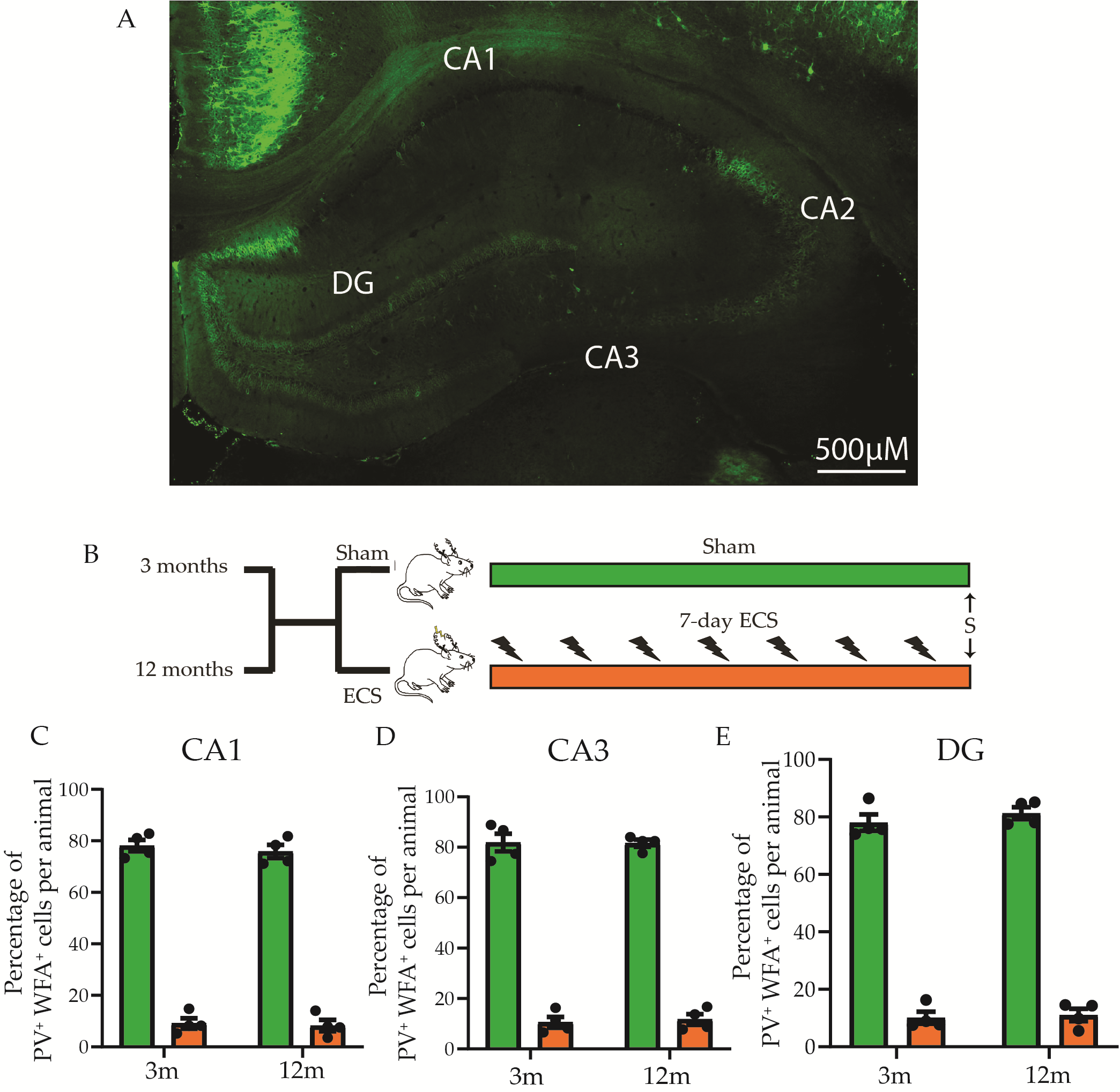

Supplement: pyad008_suppl_Supplementary_Figures [file pyad008_suppl_supplementary_figures.docx]
