# Supplementary material for: Influence of Chronic Electroconvulsive Seizures on Plasticity-Associated Gene Expression and Perineuronal Nets Within the Hippocampi of Young Adult and Middle-Aged Sprague-Dawley Rats
Source: Int J Neuropsychopharmacol. 2023 Mar 4;26(4):294–306. doi: 10.1093/ijnp/pyad008 (PMC10109107; doi:10.1093/ijnp/pyad008)
Supplement: pyad008_suppl_Supplementary_Table_S1 [file pyad008_suppl_supplementary_table_s1.docx]

| **Gene Name** | **Forward Primer (5' - 3')** | **Reverse Primer (5' - 3')** |
| --- | --- | --- |
| Acan | CCTCAGAGGTGAATGTTACCG | TGGAGAAGCAAGGGTAGGG |
| Adamts1 | AAGGCAAACGAGTCCGCTAC | GCATACTTGGGTGTCCATTCC |
| Adamts3 | ACTACCAGAATACCAAACACCA | TTCACACACTCTCCTCGCAC |
| Adamts4 | GCCTACAACCACCGAACCGA | CCTGGACACAGACAGAGGAAG |
| Adamts5 | ACCAAGAAGTTGCCTGCCGT | CAAGAGCGAGAACACTGACCC |
| Adamts9 | TGGGAAGACGGAGAAGGTAGA | GGACATCATTGCGGGTGTTG |
| Apoer2 | TTCACATAGGGAGGACGGCT | GAGGGTTCTTCGGGAGTTGG |
| Arc | GCCCCCAGCAGTGATTCATA | GACTCAGCCCCTCTGGGAC |
| Bdnf ex 1 | AGGGCAGTTGGACAGTCATTGGTA | TTCAACTCTCATCCACCTTGGCGA |
| Bdnf ex 3 | CATTGAGTCGCTGAAGTTGGCTT | GTTAACCCAGTATACCAACCCGGA |
| Bdnf ex 4 | AGGCTTTGATGAGACCGGGTT | TCACATTGTTGTCACGTCCTGGT |
| Chst11 | GAATTTGCCGGATGGTGCTGG | TGGTGCAGGATGGCAGTGTT |
| Chst3 | AGGATTGCCGGGACCTTGTG | CGATAAGAGCAGGGCTGGGT |
| Cspg4 | CTGGAGAGAGGTGGAAGAGCAG | AACAGGGAGGATGGTGATAGTG |
| Dab1 | GACTTCTACCACACCATCTACC | TCTTCACTCTTGCTGGGACTTT |
| Egr2 | CTGCCTGACAGCCTCTACCC | ATGCCATCTCCAGCCACTCC |
| Egr3 | ACTCGGTAGCCCATTACACTCAG | GTAGGTCACGGTCTTGTTGCC |
| Fgf2 | AGCGGCTCTACTGCAAGAAC | GTTCGCACACACTCCCTTG |
| Flt1 | CGGCAGACCAATACAATCCT | ACAAGAGTTTGACCACGGAGG |
| Fos | TGAAGACCATGTCAGGCGG | TTCCCTTCGGATTCTCCGT |
| Hapln1 | ACCAGGATGCTGTGATTGC | TCCCAGAACCCGTAGTTCC |
| Has2 | GCAGGAGCTGAACAAGATGC | TTGGATGATGAGGTGTGAGG |
| Homer1a | CTTCAGTCTCCTTTAACACC | TCCATATTTATCCATCTCATTT |
| Homer1b | GGGTGTCTGGAGTTCTTCCC | GCTGCCTTTGTTTAGTTGGCT |
| Hprt1 | GCAGACTTTGCTTTCCTTGG | GTCTGGCCTGTATCCAACACT |
| Igf2 | ACGCACCCGCAGAGAAATAA | CCGAGCACCTTCCTAACACC |
| Mmp2 | CCAGAGACTGCTATGTCCACT | ACACCACACCTTGCCATCG |
| Mmp9 | CATCTGTATGGTCGTGGCTCT | CTGTCGGCTGTGGTTCAG |
| Ncan | CCTTCCTCCCTCTCAATTCC | CCAAGACCAAAGACCAGAGC |
| Ngfr | AGGTGTGCAGATGTGCCTATGGCTA | ACGTGGTTGGCTTCGTCTGAGTAT |
| Ntf3 | TGCAGAGCATAAGAGTCACC | AAGTCAGTGCTCGGACGTAG |
| Ntrk2 | TTCCGAGGTTGGAACCTAACAGCA | TTGCCGTTCTTCAGAAACGCCTTG |
| pan Bdnf l | TCTTGCTGTGGTCTCTTTTTGG | CCACAGACATTTACTTACAGTTTCAATG |
| pan Bdnf s | GGCTGACACTTTTGAGCACG | CCCGGGAAGTGTACAAGTCC |
| Reln | GTCTACCTTCCACTCGCCAC | AGTCACAAATGCCTCGTCCT |
| Timp1 | CTTCCTGGTTCCCTGGCATA | ATCGCTCTGGTAGCCCTTCT |
| Timp4 | ACTCTTCTCTCTGTGGTGTGA | GCATAGCAAGTGGTGATTTGGC |
| Vcan | CAACCTTGCCCACCTTACC | TGCGTAGGCACTGATACCC |
| Vegfa | TCCAATTGAGACCCTGGTGGACAT | TCTCCTATGTGCTGGCTTTGGTGA |
| Vldr | GTCAACACAACAGACATCCTAC | TGCCATCACTAAGAGCAAGAGA |
