# Supplementary material for: Influence of Chronic Electroconvulsive Seizures on Plasticity-Associated Gene Expression and Perineuronal Nets Within the Hippocampi of Young Adult and Middle-Aged Sprague-Dawley Rats
Source: Int J Neuropsychopharmacol. 2023 Mar 4;26(4):294–306. doi: 10.1093/ijnp/pyad008 (PMC10109107; doi:10.1093/ijnp/pyad008)
Supplement: pyad008_suppl_Supplementary_Table_S2 [file pyad008_suppl_supplementary_table_s2.docx]

| Name of the experiment | Statistical tool used | *p* value | | | | *p* value summary |
| --- | --- | --- | --- | --- | --- | --- |
| Forced Swim Test (Time spent Immobile; 3m; Sham vs ECS | Unpaired *t* test  (Two tailed) | 0.0024 | | | | * |
| Forced Swim Test (Time spent Immobile; 12m; Sham vs ECS | Unpaired *t* test  (Two tailed) | 0.0012 | | | | * |
| Forced Swim Test (Time spent swimming 3m; Sham vs ECS | Unpaired *t* test  (Two tailed) | 0.1277 | | | | ns |
| Forced Swim Test (Time spent swimming 12m; Sham vs ECS | Unpaired *t* test  (Two tailed) | 0.0324 | | | | * |
| BrdU^+^ cells in the dorsal hippocampus | Two-way ANOVA with Bonferroni multiple comparisons test | Interaction | F (1,17) = 58.78;  *p* < 0.0001 | | | * |
|  |  | ECS | F (1, 17) = 96.47;  *p* < 0.0001 | | | * |
|  |  | Age | F (1,17) = 108.6;  *p* < 0.0001 | | | * |
|  | 3m Sham vs 3m ECS | *p* < 0.0001 | | | | * |
|  | 12m Sham vs 12m ECS | *p* = 0.7017 | | | | ns |
|  | 3m Sham vs 12m Sham | *p >* 0.9999 | | | | ns |
|  | 3m ECS vs 12m ECS | *p* < 0.0001 | | | | ns |
| BrdU^+^ cells in the ventral hippocampus | Two-way ANOVA with Bonferroni multiple comparisons test | Interaction | | | F (1,17) = 61.41;  *p* < 0.0001 | * |
|  |  | ECS | | | F (1, 17) = 92.4;  *p* < 0.0001 | * |
|  |  | Age | | | F (1,17) = 156.2;  *p* < 0.0001 | * |
|  | 3m Sham vs 3m ECS | *p* < 0.0001 | | | | * |
|  | 12m Sham vs 12m ECS | p = 0.7017 | | | | ns |
|  | 3m Sham vs 12m Sham | *p* < 0.0001 | | | | * |
|  | 3m ECS vs 12m ECS | *p* < 0.0001 | | | | * |
| Sox2^+^ GFAP^+^ cells in the dorsal dentate gyrus of the hippocampus | Two-way ANOVA with Bonferroni multiple comparisons test | Interaction | | | F (1,8) = 1.885;  *p* = 0.2070 | ns |
|  |  | ECS | | | F (1, 8) = 34.66;  *p =* 0.0004 | * |
|  |  | Age | | | F (1,8) = 109.6;  *p* < 0.0001 | * |
|  | 3m Sham vs 3m ECS | *p* = 0.0002 | | | | * |
|  | 12m Sham vs 12m ECS | *p* = 0.0012 | | | | * |
|  | 3m Sham vs 12m Sham | *p* = 0.0765 | | | | ns |
|  | 3m ECS vs 12m ECS | *p* = 0.0054 | | | | * |
| Reelin^+^ cells in the *Stratum oriens* - CA1 region of Hippocampus | Two-way ANOVA with Bonferroni multiple comparisons test | Interaction | | | F (1,16) = 9.768;  *p* = 0.0065 | * |
|  |  | ECS | | | F (1, 16) = 13.2;  *p =* 0.0022 | * |
|  |  | Age | | | F (1,16) = 0.709;  *p* = 0.4121 | ns |
|  | 3m Sham vs 3m ECS | *p* = 0.0004 | | | | * |
|  | 12m Sham vs 12m ECS | *p* > 0.9999 | | | | ns |
|  | 3m Sham vs 12m Sham | *p* = 0.0358 | | | | * |
|  | 3m ECS vs 12m ECS | *p* = 0.7559 | | | | ns |
| Reelin^+^ cells in the *Stratum pyramidale* - CA1 region of Hippocampus | Two-way ANOVA with Bonferroni multiple comparisons test | Interaction | | | F (1,16) = 4.902;  *p* = 0.0417 | * |
|  |  | ECS | | | F (1, 16) = 12.4;  *p =* 0.0028 | * |
|  |  | Age | | | F (1,16) = 0.155;  *p* = 0.6955 | ns |
|  | 3m Sham vs 3m ECS | *p* = 0.0020 | | | | * |
|  | 12m Sham vs 12m ECS | *p* > 0.9999 | | | | ns |
|  | 3m Sham vs 12m Sham | *p* = 0.4996 | | | | ns |
|  | 3m ECS vs 12m ECS | *p* > 0.9999 | | | | ns |
| Reelin^+^ cells in the *Stratum oriens* – CA3 region of Hippocampus | Two-way ANOVA | Interaction | | | F (1,16) = 0.241;  *p* = 0.6296 | ns |
|  |  | ECS | | | F (1, 16) = 0.14;  *p =* 0.7044 | ns |
|  |  | Age | | | F (1,16) = 1.610;  *p* = 0.2227 | ns |
| Reelin^+^ cells in the *Stratum pyramidale* – CA3 region of Hippocampus | Two-way ANOVA | Interaction | | | F (1,16) = 0.056;  *p* = 0.8159 | ns |
|  |  | ECS | | | F (1, 16) = 0.03;  *p =* 0.8478 | ns |
|  |  | Age | | | F (1,16) = 6.341;  *p* = 0.0228 | * |
| Reelin^+^ cells in the dentate gyrus region of Hippocampus | Two-way ANOVA with Bonferroni multiple comparisons test | Interaction | | | F (1,16) = 15.65;  *p* = 0.0011 | * |
|  |  | ECS | | | F (1, 16) = 21.7;  *p =* 0.0003 | * |
|  |  | Age | | | F (1,16) = 5.184;  *p* = 0.0369 | * |
|  | 3m Sham vs 3m ECS | *p* < 0.0001 | | | | * |
|  | 12m Sham vs 12m ECS | *p* > 0.9999 | | | | ns |
|  | 3m Sham vs 12m Sham | *p* > 0.9999 | | | | ns |
|  | 3m ECS vs 12m ECS | *p* = 0.0026 | | | | * |
| Reelin^+^ cells in the hilus region of Hippocampus | Two-way ANOVA | Interaction | | F (1,16) = 2.306;  *p* = 0.1484 | | ns |
|  |  | ECS | | F (1, 16) = 4.59;  *p =* 0.0478 | | * |
|  |  | Age | | F (1,16) = 1.831;  *p* = 0.1948 | | ns |
| Reelin^+^ cells in the hippocampal fissure region | Two-way ANOVA with Bonferroni multiple comparisons test | Interaction | | F (1,16) = 8.526;  *p* = 0.0100 | | * |
|  |  | ECS | | F (1, 16) = 13.5;  *p =* 0.0020 | | * |
|  |  | Age | | F (1,16) = 0.097;  *p* = 0.7591 | | ns |
|  | 3m Sham vs 3m ECS | *p* = 0.0005 | | | | * |
|  | 12m Sham vs 12m ECS | *p* > 0.9999 | | | | ns |
|  | 3m Sham vs 12m Sham | *p* = 0.2177 | | | | ns |
|  | 3m ECS vs 12m ECS | *p* = 0.5026 | | | | ns |
| WFA^+^ cells in the CA1 region of the hippocampus | Two-way ANOVA | Interaction | | F (1,19) = 3.960;  *p* = 0.0612 | | ns |
|  |  | ECS | | F (1, 19) = 42.2;  *p* < 0.0001 | | * |
|  |  | Age | | F (1,19) = 0.199;  *p* = 0.6600 | | ns |
| WFA^+^ cells in the CA3 region of the hippocampus | Two-way ANOVA with Bonferroni multiple comparisons test | Interaction | | F (1,19) = 4.714;  *p* = 0.0428 | | * |
|  |  | ECS | | F (1, 19) = 53.1;  *p* < 0.0001 | | * |
|  |  | Age | | F (1,19) = 0.926;  *p* = 0.3478 | | ns |
|  | 3m Sham vs 3m ECS | *p* < 0.0001 | | * | |  |
|  | 12m Sham vs 12m ECS | *p* = 0.0132 | | * | |  |
|  | 3m Sham vs 12m Sham | *p* = 0.2099 | | ns | |  |
|  | 3m ECS vs 12m ECS | *p* > 0.9999 | | ns | |  |
| WFA^+^ cells in the dentate gyrus of the hippocampus | Two-way ANOVA | Interaction | | F (1,19) = 0.962;  *p* = 0.3390 | | ns |
|  |  | ECS | | F (1, 19) = 12.6;  *p* = 0.0021 | | * |
|  |  | Age | | F (1,19) = 9.895;  *p* = 0.0053 | | * |
| PV+ WFA+ cells in the CA1 region of the hippocampus | Two-way ANOVA | Interaction | | F (1,12) = 0.098;  *p* = 0.7593 | | ns |
|  |  | ECS | | F (1, 12) = 911;  *p* < 0.0001 | | * |
|  |  | Age | | F (1,12) = 0.481;  *p* = 0.5012 | | ns |
| PV+ WFA+ cells in the CA3 region of the hippocampus | Two-way ANOVA | Interaction | | F (1,12) = 0.080;  *p* = 0.7818 | | ns |
|  |  | ECS | | F (1, 12) = 851;  *p* < 0.0001 | | * |
|  |  | Age | | F (1,12) = 0.031;  *p* = 0.8622 | | ns |
| PV+ WFA+ cells in the dentate gyrus of the hippocampus | Two-way ANOVA | Interaction | | F (1,12) = 0.252;  *p* = 0.6243 | | ns |
|  |  | ECS | | F (1, 12) = 900;  *p* < 0.0001 | | * |
|  |  | Age | | F (1,12) = 0.832;  *p* = 0.3796 | | ns |
