# Supplementary material for: Influence of Chronic Electroconvulsive Seizures on Plasticity-Associated Gene Expression and Perineuronal Nets Within the Hippocampi of Young Adult and Middle-Aged Sprague-Dawley Rats
Source: Int J Neuropsychopharmacol. 2023 Mar 4;26(4):294–306. doi: 10.1093/ijnp/pyad008 (PMC10109107; doi:10.1093/ijnp/pyad008)
Supplement: pyad008_suppl_Supplementary_Table_S3 [file pyad008_suppl_supplementary_table_s3.docx]

Rationale for selection of genes to be profiled following chronic ECS in young adult and middle-aged Sprague Dawley Rats

| **Broad Gene Function** | **Gene Name** | **Physiological role** | **Activity dependent regulation** |
| --- | --- | --- | --- |
| Immediate Early Genes (IEG) | Arc, Egr2/3, cFos, Homer1a/1b, BDNF | Rapid regulation in response to neuronal activity, Influence on gene expression, cytoarchitecture, trophic action and synaptogenesis | (Cole et al., 1990; Bramham et al., 2008; Gallitano-Mendel et al., 2008; Calais et al., 2013; Leal et al., 2017; Carmichael and Henley, 2018; Meyers et al., 2018; Clifton et al., 2019) |
| Transcription Factors | Egr2/Egr3, cFos | Driving of both early and late response gene regulation | (Cole et al., 1990; Gallitano-Mendel et al., 2008; Calais et al., 2013; Meyers et al., 2018) |
| Cytoskeletal associated proteins | Arc, Homer1a/1b | Dendritogenesis, axon genesis, synapse stabilization, synaptic pruning | (Winston et al., 1990; Schmoll et al., 2001; Bramham et al., 2008; Calais et al., 2013; Carmichael and Henley, 2018; Clifton et al., 2019) |
| Growth Factors & Neurotrophins | BDNF, Ntrk2, Ngfr, Vegfa, Flt1, Fgf2, Igf2, Nt3 | Neuronal survival, neuritogenesis, synaptic strength, synaptic plasticity, memory formation | (Nibuya et al., 1995; Gwinn et al., 2002; Pae et al., 2008; Segi-Nishida et al., 2008; Lau et al., 2010; Chapleau and Pozzo-Miller, 2012; Stern et al., 2014; Leal et al., 2017; Casarotto et al., 2021) |
| Reelin pathway associated genes | Apoer2, Reln, Dab1, Vldr | Synaptic plasticity, long-term memory, | (Weeber et al., 2002; Herz and Chen, 2006; Trotter et al., 2013; Dlugosz and Nimpf, 2018) |
| Perineuronal Structure and Synthesis associated genes | Acan, Cspg4, Chst3, Chst11, Hapln1, Has2, Ncan, Vcan | Formation of PNN, Plasticity modulation, buffer oxidative stress, closing of critical period plasticity | (McRae et al., 2012; Geissler et al., 2013; Guirado et al., 2014; Carstens et al., 2016; Gottschling et al., 2019) |
| Perineuronal dissolution | Adamts1, 3, 4, 5, 9, Mmp2, Mmp9, Timp1, Timp4 | Reopening of critical period plasticity | (Benekareddy et al., 2008; McRae et al., 2012; Guirado et al., 2014; Dubey et al., 2017) |

References:

- Benekareddy M, Mehrotra P, Kulkarni VA, Ramakrishnan P, Dias BG, Vaidya VA (2008) Antidepressant treatments regulate matrix metalloproteinases-2 and -9 (MMP-2/MMP-9) and tissue inhibitors of the metalloproteinases (TIMPS 1-4) in the adult rat hippocampus. Synapse 62:590–600.
- Bramham CR, Worley PF, Moore MJ, Guzowski JF (2008) The immediate early gene arc/arg3.1: regulation, mechanisms, and function. J Neurosci Off J Soc Neurosci 28:11760–11767.
- Calais JB, Valvassori SS, Resende WR, Feier G, Athié MCP, Ribeiro S, Gattaz WF, Quevedo J, Ojopi EB (2013) Long-term decrease in immediate early gene expression after electroconvulsive seizures. J Neural Transm 120:259–266.
- Carmichael RE, Henley JM (2018) Transcriptional and post-translational regulation of Arc in synaptic plasticity. Semin Cell Dev Biol 77:3–9.
- Carstens KE, Phillips ML, Pozzo-Miller L, Weinberg RJ, Dudek SM (2016) Perineuronal Nets Suppress Plasticity of Excitatory Synapses on CA2 Pyramidal Neurons. J Neurosci Off J Soc Neurosci 36:6312–6320.
- Casarotto PC et al. (2021) Antidepressant drugs act by directly binding to TRKB neurotrophin receptors. Cell 184:1299-1313.e19.
- Chapleau CA, Pozzo-Miller L (2012) Divergent roles of p75NTR and Trk receptors in BDNF’s effects on dendritic spine density and morphology. Neural Plast 2012:578057.
- Clifton NE, Trent S, Thomas KL, Hall J (2019) Regulation and Function of Activity-Dependent Homer in Synaptic Plasticity. Mol neuropsychiatry 5:147–161.
- Cole AJ, Abu‐Shakra S, Saffen DW, Baraban JM, Worley PF (1990) Rapid Rise in Transcription Factor mRNAs in Rat Brain After Electroshock‐Induced Seizures. J Neurochem 55.
- Dlugosz P, Nimpf J (2018) The Reelin Receptors Apolipoprotein E receptor 2 (ApoER2) and VLDL Receptor. Int J Mol Sci 19.
- Dubey D, McRae PA, Rankin-Gee EK, Baranov E, Wandrey L, Rogers S, Porter BE (2017) Increased metalloproteinase activity in the hippocampus following status epilepticus. Epilepsy Res 132:50–58.
- Gallitano-Mendel A, Izumi Y, Tokuda K, Zorumski CF, Howell MP, Muglia LJ, Wozniak DF, Milbrandt JD (2008) The immediate early gene Egr3 mediates adaptation to stress and novelty.
- Geissler M, Gottschling C, Aguado A, Rauch U, Wetzel CH, Hatt H, Faissner A (2013) Primary hippocampal neurons, which lack four crucial extracellular matrix molecules, display abnormalities of synaptic structure and function and severe deficits in perineuronal net formation. J Neurosci Off J Soc Neurosci 33:7742–7755.
- Gottschling C, Wegrzyn D, Denecke B, Faissner A (2019) Elimination of the four extracellular matrix molecules tenascin-C, tenascin-R, brevican and neurocan alters the ratio of excitatory and inhibitory synapses. Sci Rep 9:13939.
- Guirado R, Perez-Rando M, Sanchez-Matarredona D, Castrén E, Nacher J (2014) Chronic fluoxetine treatment alters the structure, connectivity and plasticity of cortical interneurons. Int J Neuropsychopharmacol 17:1635–1646.
- Gwinn RP, Kondratyev A, Gale K (2002) Time-dependent increase in basic fibroblast growth factor protein in limbic regions following electroshock seizures. Neuroscience 114:403–409 Available at: https://www.sciencedirect.com/science/article/pii/S0306452202002658.
- Herz J, Chen Y (2006) Reelin, lipoprotein receptors and synaptic plasticity. Nat Rev Neurosci 7:850–859.
- Lau AG, Irier HA, Gu J, Tian D, Ku L, Liu G, Xia M, Fritsch B, Zheng JQ, Dingledine R, Xu B, Lu B, Feng Y (2010) Distinct 3’UTRs differentially regulate activity-dependent translation of brain-derived neurotrophic factor (BDNF). Proc Natl Acad Sci U S A 107:15945–15950.
- Leal G, Bramham CR, Duarte CB (2017) BDNF and Hippocampal Synaptic Plasticity. Vitam Horm 104:153–195.
- McRae PA, Baranov E, Rogers SL, Porter BE (2012) Persistent decrease in multiple components of the perineuronal net following status epilepticus. Eur J Neurosci 36:3471–3482.
- Meyers KT, Marballi KK, Brunwasser SJ, Renda BA, Charbel M, Marrone DF, Gallitano AL (2018) The Immediate Early Gene Egr3 Is Required for Hippocampal Induction of Bdnf by Electroconvulsive Stimulation. Front Behav Neurosci 12.
- Nibuya M, Morinobu S, Duman RS (1995) Regulation of BDNF and trkB mRNA in rat brain by chronic electroconvulsive seizure and antidepressant drug treatments. J Neurosci 15:7539–7547.
- Pae C-U, Marks DM, Han C, Patkar AA, Steffens D (2008) Does neurotropin-3 have a therapeutic implication in major depression? Int J Neurosci 118:1515–1522.
- Schmoll H, Badan I, Fischer B, Wagner AP (2001) Dynamics of gene expression for immediate early- and late genes after seizure activity in aged rats. Arch Gerontol Geriatr 32:199–218 Available at: https://www.sciencedirect.com/science/article/pii/S0167494301001017.
- Segi-Nishida E, Warner-Schmidt JL, Duman RS (2008) Electroconvulsive seizure and VEGF increase the proliferation of neural stem-like cells in rat hippocampus. Proc Natl Acad Sci U S A 105:11352–11357.
- Stern SA, Chen DY, Alberini CM (2014) The effect of insulin and insulin-like growth factors on hippocampus- and amygdala-dependent long-term memory formation. Learn Mem 21:556–563.
- Trotter J, Lee GH, Kazdoba TM, Crowell B, Domogauer J, Mahoney HM, Franco SJ, Müller U, Weeber EJ, D’Arcangelo G (2013) Dab1 is required for synaptic plasticity and associative learning. J Neurosci Off J Soc Neurosci 33:15652–15668.
- Weeber EJ, Beffert U, Jones C, Christian JM, Forster E, Sweatt JD, Herz J (2002) Reelin and ApoE receptors cooperate to enhance hippocampal synaptic plasticity and learning. J Biol Chem 277:39944–39952.
- Winston SM, Hayward M, Nestler EJ, Duman RS (1990) Chronic Electroconvulsive Seizures Down–Regulate Expression of the Immediate‐Early Genes c‐fos and c‐jun in Rat Cerebral Cortex. J Neurochem 54.
